# Twelve Years of Rabies Surveillance in Sri Lanka, 1999–2010

**DOI:** 10.1371/journal.pntd.0003205

**Published:** 2014-10-09

**Authors:** Dushantha Karunanayake, Takashi Matsumoto, Omala Wimalaratne, Susilakanthi Nanayakkara, Devika Perera, Akira Nishizono, Kamruddin Ahmed

**Affiliations:** 1 Department of Rabies Diagnosis and Research, Medical Research Institute, Colombo, Sri Lanka; 2 Department of Microbiology, Faculty of Medicine, Oita University, Yufu, Japan; 3 Research Promotion Institute, Oita University, Yufu, Japan; The Global Alliance for Rabies Control, United States of America

## Abstract

**Background:**

Rabies is endemic in Sri Lanka, but little is known about the temporal and spatial trends of rabies in this country. Knowing these trends may provide insight into past control efforts and serve as the basis for future control measures. In this study, we analyzed distribution of rabies in humans and animals over a period of 12 years in Sri Lanka.

**Methods:**

Accumulated data from 1999 through 2010 compiled by the Department of Rabies Diagnosis and Research, Medical Research Institute (MRI), Colombo, were used in this study.

**Results:**

The yearly mean percentage of rabies-positive sample was 62.4% (47.6–75.9%). Three-fourths of the rabies-positive samples were from the Colombo, Gampaha, and Kalutara districts in Western province, followed by Galle in Southern province. A high percentage of the rabies samples were from dogs (85.2%), followed by cats (7.9%), humans (3.8%), wild animals (2.0%), and livestock (1.1%). Among wild animals, mongooses were the main victims followed by civets. The number of suspect human rabies cases decreased gradually in Sri Lanka, although the number of human samples submitted for laboratory confirmation increased.

**Conclusions:**

The number of rabid dogs has remained relatively unchanged, but the number of suspect human rabies is decreasing gradually in Sri Lanka. These findings indicate successful use of postexposure prophylaxis (PEP) by animal bite victims and increased rabies awareness. PEP is free of charge and is supplied through government hospitals by the Ministry of Health, Sri Lanka. Our survey shows that most positive samples were received from Western and Southern provinces, possibly because of the ease of transporting samples to the laboratory. Submissions of wild animal and livestock samples should be increased by creating more awareness among the public. Better rabies surveillance will require introduction of molecular methods for detection and the establishment of more regional rabies diagnostic laboratories.

## Author Summary

Rabies is a public health concern in Sri Lanka. The incidence of dog rabies remains unchanged, but the incidence of suspect human rabies is decreasing gradually in Sri Lanka. This finding indicates the effects of improved access to postexposure prophylaxis by animal bite victims and increased rabies awareness. As in other rabies-endemic countries, in Sri Lanka, human rabies is transmitted mainly by dogs, although domestic and wild animals have been diagnosed rabid, and can pose a risk of exposure to humans. In this study, we analyzed 12 years of data accumulated in the national reference laboratory of Sri Lanka to identify the trends of rabies in this country. This study showed that rabies has been recorded mainly in Western and Southern Provinces of Sri Lanka, possibly because of the ease of communication with rabies diagnostic laboratories from these areas. Regional rabies diagnosis laboratories should be established to improve surveillance of rabies in Sri Lanka. There were few submitted animal samples from livestock and wild animals, indicating that greater awareness is needed among the public regarding the need to submit suspect rabid animals for diagnostic evaluation. These data could help policy makers improve rabies prevention and to control rabies in Sri Lanka.

## Introduction

Each year 55,000 people die of rabies throughout the world, more than 31,000 of these deaths occur in Asia [1], [2]. Sri Lanka is one Asian country where human deaths from rabies has decreased markedly during the past decade [3]; however, rabies is endemic and remains a significant public health problem in this country. Sri Lanka is a tropical island state situated in the Indian Ocean near the southern tip of India. The current population is about 20.2 million of this 18.3% living in urban areas, 77.3% in rural areas, and 4.4% living in estate areas (http://www.statistics.gov.lk). According to the Census Ordinance ‘Estate” has been defined as areas with plantations where there are 20 or more acres in land and 10 or more resident laborers. Sri Lanka is one of the fastest growing economies in Asia and only 4.3% of the population is living under poverty line. The Sri Lankan Ministry of Health spends a substantial amount of its health budget on anti-rabies treatment for humans. Recent estimates that the cost of post-exposure prophylaxis (PEP) per patient is US$173 without immunoglobulin and US$177 with equine immunoglobulin [4]. This includes all direct medical costs associated with PEP. As in other canine rabies-endemic countries, dogs are the main transmitter of rabies to humans in Sri Lanka [5].Recently, the confirmation of sylvatic rabies virus in a civet submitted to Medical Research Institute (MRI) for diagnosis from Moneragala district [6] sparked discussion about whether there are other reservoir species for sylvatic rabies in Sri Lanka and whether these animals are widespread throughout the country or localized to a particular area. To explore other reservoir species of rabies viruses in Sri Lanka, the epidemiology of rabies virus variants must be analyzed in different animals and their geographical locations recorded. The rabies endemic regions may expand or contract as a result of virus transmission and animal population interactions [7]. Therefore a long term study focusing on the molecular epidemiology of rabies in Sri Lankan would provide a better understanding of the epidemiology of rabies throughout the country.

Among several factors, natural and anthropometric factors have a direct impact on animal population dynamics [8], and geographic features may act as barriers or corridors for the spread of rabies [9]. Additionally, unusual animal-dispersal patterns and human-mediated translocation of infected animals have resulted in the unexpected introduction of rabies into new areas [8]. Occasionally, transmission of rabies virus variants to new hosts can perpetuate regionally, and these variants can become enzootic in new reservoir species [8]. All mammals are susceptible to rabies, but some animal species do not serve as a reservoir species for rabies virus and therefore do not normally play a role in transmission of the virus. A clearer understanding of the reservoir species for rabies would help to improve rabies prevention and control in Sri Lanka, a country rich in animal diversity.

In recent years, Sri Lanka has experienced population growth, rapid urbanization, deforestation, and construction of new highways, dams, and irrigation systems. All of these changes can affect reservoir species habitat and may influence the epidemiology of rabies in different ways. This study was performed to identify the trends of rabies infection in Sri Lanka during the past 12 years by analyzing the number of rabies specimens received at the MRI, the national rabies laboratory in the country. We expect that the findings will be useful for formulating appropriate strategies to strengthen rabies control activities in Sri Lanka.

## Methods

### Data collection and analyses

**Figure 1:**
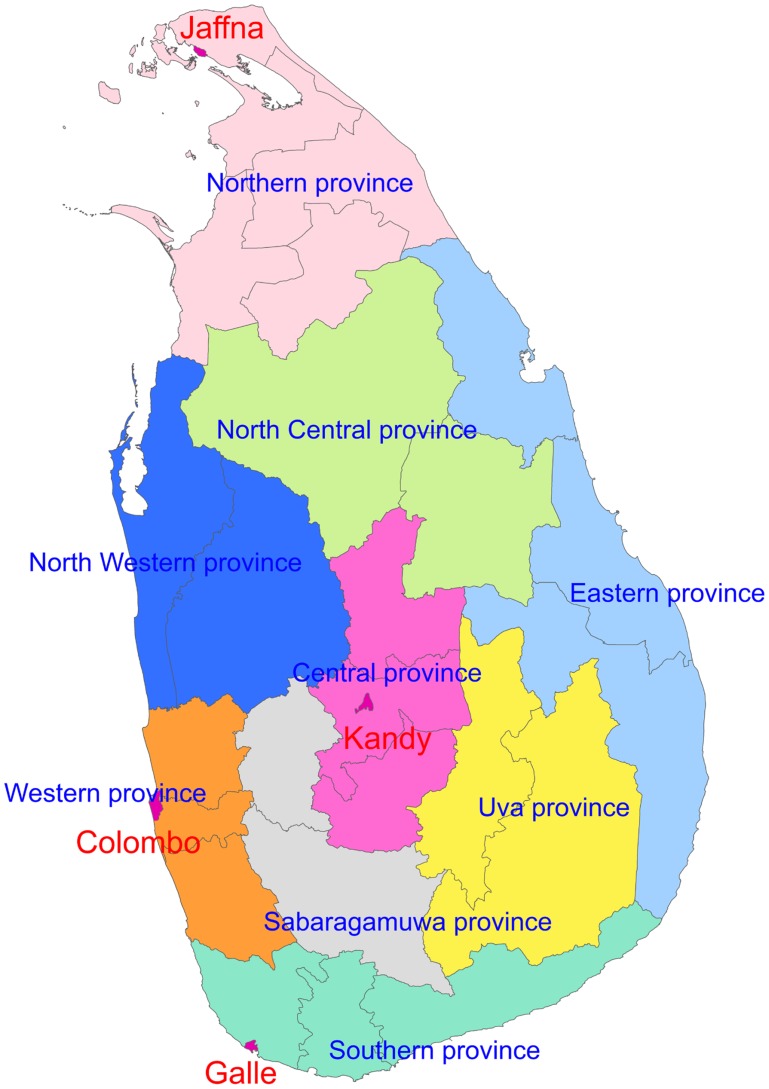
A map showing provinces and major cities in Sri Lanka.

The study used accumulated data from 1999 through 2010 collected by the Department of Rabies Diagnosis and Research, Medical Research Institute (MRI), Colombo, which is the national reference laboratory for human and animal rabies diagnosis in Sri Lanka (Figure 1). There are two regional rabies diagnostic laboratories in Peradeniya and Karapitiya for testing animal samples only. The Peradeniya laboratory was established in 2007 and is attached to the Faculty of Veterinary Medicine and Animal Science, University of Peradeniya. Peradeniya has about 50,000 inhabitants and is a suburb of Kandy the administrative capital of Central province. The Karapitiya laboratory is attached to the Karapitiya teaching hospital, Karapitiya, Galle. Galle is the administrative capital of Southern province and has a population of about 90,000. During the tsunami in December 2004, the Karapitiya laboratory was destroyed, therefore all samples from Galle were sent to MRI. The Karapitiya laboratory is currently functioning modestly after reconstruction and is gradually increasing its capacity to commence on the florescence antibody test (FAT). Before the tsunami and after the reconstruction, samples that were reported as having inconclusive results, and samples that were reported as negative but clinically were suggestive of rabies were sent from Karapitiya laboratory to MRI for confirmation. A number of FAT inconclusive samples were also sent from Peradeniya laboratory to the MRI for confirmation of the results. Therefore a large number of samples sent from Karapitiya and Peradeniya to the MRI were included in this study. From the regional laboratories, brain samples were transported in ice box. Therefore, the samples received at the MRI were representative for all of Sri Lanka.

Animal rabies is not a notifiable disease in Sri Lanka. For animal samples, the head of the suspected animal was submitted to the laboratory, and the brain was dissected by a trained person. Animals submitted for testing were exhibiting clinical signs suggestive of rabies with or without a history of biting humans and/or animals. The clinical signs of rabies included aggressive behavior, biting tendency, and/or excessive salivation. Samples from animals suspected of being rabid are usually submitted by general public, a small number of samples are also submitted by veterinarians and forest rangers. There are no community veterinarians in Sri Lanka. To evaluate the correlation between the mean yearly numbers of rabies positive samples in different provinces and their population or population density of 2012, the Pearson correlation test was performed.

Human rabies is notifiable in Sri Lanka. Human brains from clinically diagnosed rabies patients were sent by the Judicial Medical Officers in their respective districts. The samples were packaged without preservative and the cold chain was maintained during transportation. FAT was used as the reference test to confirm the diagnosis of rabies in all human specimens and was performed immediately after the samples were received [10].

In the MRI, in the analysis of animal and human brains, FAT is performed on crushed smears of hippocampus and brain stem, respectively. FAT is performed free of charge and test results are reported by hard copy to the person who submitted the sample. In the case of an emergency, such as when PEP is required, the patient is informed by telephone. Annual data are also reported to the Ministry of Health. All brain samples that tested positive by FAT for the presence of rabies virus were reported to the authorities as being from a rabies infected animal. Similarly, when a human brain sample from a suspected rabies case was positive for rabies by FAT that patient was designated as a laboratory confirmed human case. The number of samples and their results were, and continue to be updated regularly in the laboratory's database. Data on human deaths from suspect rabies were collected from the website of the Public Health Veterinary Services, Ministry of Health, Sri Lanka (www.rabies.gov.lk). A suspect rabies case was defined as having agitation, hydrophobia, aerophobia, photophobia, or hypersalivation plus a history of animal bite. To evaluate the correlation between the number of suspect human rabies deaths and Sri Lankan government's annual expenditure on PEP, the Pearson correlation test was performed. Data on the financial expenditure for PEP was obtained from the Medical Supplies Division, Ministry of Health, Sri Lanka. Data were available from 2005. ArcGIS 10.0 software [Environmental Systems Research Institute, Inc. (ESRI), USA] was used to illustrate surveyed areas in different districts of Sri Lanka.

### Ethics statement

The institutional review committee of the MRI approved the study. The data used in this study were anonymized.

## Results

During the 12 years, a total of 12,835 samples were received at the MRI, 7,815 of these samples were FAT positive. Each year, a mean (range) 1069.5 (734–1367) samples were submitted, 651.2 (482–819) were FAT positive (Table 1). The yearly mean percentage of positive samples was 62.4% (47.6–75.9%).

**Table 1:** Number of rabies positive and negative samples among the total number of samples received at the Medical Research Institute, Sri Lanka between 1999 and 2010.

Samples	1999	2000	2001	2002	2003	2004	2005	2006	2007	2008	2009	2010	Total
Total	925	764	734	681	885	1045	1118	1336	1306	1367	1313	1361	12835
Positive	702	574	516	482	550	646	543	819	753	791	791	648	7815
Negatives	223	190	218	199	335	399	575	517	553	576	522	713	5020
Human	20	21	26	27	31	38	27	42	35	37	46	33	383
Positive	18 (90%)	15 (71.4%)	17 (65.4%)	17 (63%)	15 (48.4%)	25 (65.8%)	20 (74.1%)	32 (76.2%)	32 (91.4%)	31 (83.8%)	42 (91.3%)	32 (97%)	296 (77.3%)
Negative	2	6	9	10	16	13	7	10	3	6	4	1	87
Animal cases	905	743	708	654	854	1007	1091	1294	1271	1330	1267	1328	12452
Positive	684 (75.6%)	559 (75.2%)	499 (70.5%)	465 (71.1%)	535 (62.6%)	621 (61.7%)	523 (47.9%)	787 (60.8%)	721 (56.7%)	760 (57.1%)	749 (59.1%)	616 (46.4%)	7519 (60.4%)
Negative	221	184	209	189	319	386	568	507	550	570	518	712	4933

Among the samples received, the animal species that comprised the highest number of positives and percent positive in decreasing order included: dogs (9,683 and 75.4%), cats (1,962 and 15.3%), wild animals (658 and 5.1%), humans (383 and 3.0%), and livestock (149 and 1.2%). Among the 7,815 rabies-positive samples, the majority were from dogs (6,662 and 85.2%), followed by cats (615 and 7.9%), humans (296 and 3.8%), wild animals (154 and 2.0%), and livestock (88 and 1.1%). The percentage of rabies-positive samples of the samples received was in the following order: humans (77.3%), livestock (71.6%), dogs (68.8%), cats (31.3%) and wild animals (14.9%) (Table 2).

**Table 2:** The percentage of rabies-positive samples in humans, dogs, cats, livestock and wildlife during 12 years.

	Total	Positive	Negative	Positive rate (%)
Humans	383	296	87	77.3
Livestock	215	154	61	71.6
Dogs	9683	6662	3021	68.8
Cats	1962	615	1347	31.3
Wildlife	592	88	504	14.9

### Animal rabies

During the 12 years of this retrospective study, a total of 12,452 samples were received at the MRI, 7,519 of which were FAT positive (Table 1). Each year an average of, 626.6 (465–787) of samples were FAT positive. There was a decreasing trend in the number of positive samples between 1999 and 2002 followed by an increase that peaked in 2006 and then decreased again. By contrast the percentage of positive samples decreased gradually until 2005(47.9%), increased in 2006 (60.8%), where it remained at approximately the same level until it decreased again in 2010 (46.4%). The yearly mean percentage of positive samples was 62.1% (47.9–75.6%).

Samples were received from 24 of 25 districts in nine provinces of Sri Lanka. When classified by district, most rabies-positive samples were submitted from Colombo, Gampaha, Galle and Kalutara (Table 3). When classified by province (Table 4), the numbers of positive samples (percentage of the total positive samples from all provinces)/the numbers of submitted samples during the 12 years originated from, 5,701 (75.8%)/9,837 Western province; 896 (11.9%)/1076 Southern province; 321 (4.3%)/553 North Western province; 291 (3.9%)/493 Sabaragamuwa province; 137 (1.8%)/227 Central province; 90 (1.2%)/146 Uva province; 49 (0.6%)/77 North Central province; 28 (0.4%)/35 Eastern province; and 6 (0.1%)/8 Northern province.

**Table 3:** The annual number of animal rabies cases in all provinces and their respective districts of Sri Lanka.

Year	Central			Western			Southern			Eastern			North					NW		NC		Sabaragamuwa		Uva		Total
	MT	KY	NW	CO	GQ	KT	GL	MH	HB	TC	APR	BC	JA	MB	VA	MP	KL	PX	KG	AD	PR	RN	KE	MJ	BD	
1999	1	7	0	306	243	56	24	6	1	2	1	0	0	0	0	0	0	6	5	1	5	12	4	0	4	684
2000	0	3	0	255	207	41	23	4	0	0	0	0	0	0	0	0	0	2	2	3	0	12	5	0	2	559
20001	1	3	0	205	206	45	19	1	3	0	0	0	0	0	0	0	0	5	3	2	1	3	1	0	1	499
2002	0	9	0	160	168	57	24	5	3	0	1	0	0	0	0	0	0	8	11	0	5	7	3	2	2	465
2003	0	12	3	258	115	37	68	10	2	1	0	0	0	0	0	0	0	8	7	0	0	8	5	1	0	535
2004	0	2	1	271	131	65	100	6	2	1	0	1	0	0	0	0	0	9	4	4	3	13	6	0	2	621
2005	2	3	1	216	131	38	69	12	5	0	1	0	0	0	0	0	0	6	5	1	3	21	5	2	2	523
2006	8	8	1	279	218	43	98	22	2	4	0	0	0	0	0	0	0	14	11	3	2	39	20	3	12	787
2007	11	12	2	223	176	65	123	9	6	1	1	1	1	0	1	0	0	31	10	2	3	19	14	3	7	721
2008	4	23	1	187	215	70	136	9	4	1	4	0	1	0	0	0	0	43	20	2	2	12	15	2	9	760
2009	8	4	2	214	221	78	70	14	9	0	2	1	2	1	0	0	0	43	18	4	2	32	9	7	8	749
2010	4	1	0	198	255	48	1	3	3	1	3	1	0	0	0	0	0	39	11	1	0	13	13	12	9	616
Total	39	87	11	2772	2286	643	755	101	40	11	13	4	4	1	1	0	0	214	107	23	26	191	100	32	58	7519

Abbreviation of provinces and districts are shown below: North Western province; NW, North Central province; NC, Ampara; APR, Anuradhapura; AD, Badulla; BD, Batticaloa; BC, Colombo; CO, Galle; GL, Gampaha; GQ, Hambantota; HB, Jaffna; JA, Kalutara; KT, Kandy; KY, Kegalle; KE, Kilinochchi; KL, Kurunegala; KG, Mannar; MB, Matale; MT, Matara; MH, Moneragala; MJ, Mullaitivu; MP, Nuwara Eliya; NW, Polonnaruwa; PR, Puttalam; PX, Ratnapura; RN, Trincomalee; TC, and Vavuniya; VA.

**Table 4:** The numbers of rabies positive and submitted samples during 12 years in all provinces.

Name of the provinces	Number of rabies positive samples/Number of submitted samples during 12 years	Percentage of the total positive samples	Mean yearly number (range) of positive samples
Western	5,701/9,837	75.8%	475.1 (385–605)
Southern	896/1076	11.9%	74.7 (7–149)
North Western	321/553	4.3%	26.7 (4–63)
Sabaragamuwa	291/493	3.9%	24.2 (4–59)
Central	137/227	1.8%	11.4 (3–28)
Uva	90/146	1.2%	7.5 (1–21)
North Central	49/77	0.6%	4.1 (0–7)
Eastern	28/35	0.4%	2.3 (0–5)
Northern	6/8	0.1%	0.5 (0–3)

The percentage of the total positive samples and the mean yearly numbers (range) of positive samples from all provinces are also shown.

The mean yearly numbers (range) of positive samples (Table 4)were found in the following order: 475.1 (385–605) from Western province, 74.7 (7–149) from Southern province, 26.7 (4–63) from North Western province, 24.2 (4–59) from Sabaragamuwa province, 11.4 (3–28) from Central province, 7.5 (1–21) from Uva province, 4.1 (0–7) from North Central province, 2.3 (0–5) from Eastern province, and 0.5 (0–3) from Northern province. The Pearson correlation coefficient value of R indicating that there is a strong correlation between the mean yearly numbers of rabies positive samples in different provinces and their population (R = 0.9458) or population density (R = 0.9759).

Livestock that were confirmed positive for rabies included pigs, cows, horses, buffalo, and goats. Wild animal species included mongooses, polecats, squirrels, foxes, monkeys, rabbits, bandicoots, wild cats, jackals, rock squirrels, and deer. The mean yearly numbers (range) of positive animal samples were 555.2 (392–704) for dog, 51.2 (28–78) for cat, 7.3 (1–17) for wild animal, and 12.8 (9–16) for livestock (Table 5).

**Table 5:** The number of rabies positive samples detected by fluorescent antibody test in samples from humans, dogs, cats, wildlife and livestock.

	1999	2000	2001	2002	2003	2004	2005	2006	2007	2008	2009	2010	Total
Humans	18	15	17	17	15	25	20	32	32	31	42	32	296
Dogs	613	486	432	392	490	567	449	704	658	681	650	540	6662
Cats	54	57	44	46	28	33	51	66	42	62	78	54	615
Wildlife	7	7	12	17	6	6	7	3	6	1	9	7	88
Livestock	10	9	11	10	11	15	16	14	15	16	12	15	154
Total	702	574	516	482	550	646	543	819	753	791	791	648	7815

**Figure 2:**
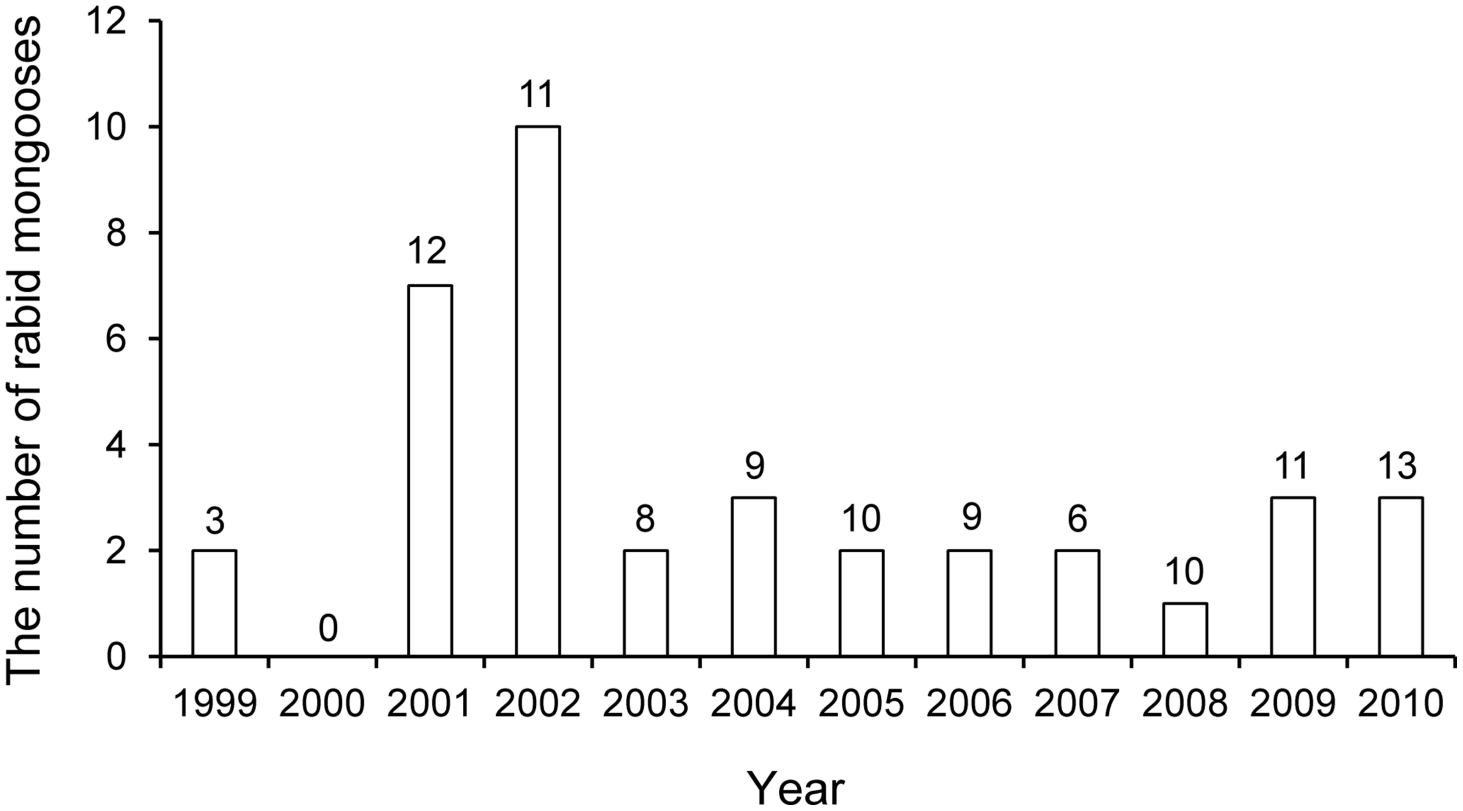
The annual number of rabies-positive samples from mongooses in Sri Lanka is shown as bar, on the background showing number of samples tested.

Among the livestock (Table 6), cows (110) had the highest number of cases of rabies followed by goats (26), buffalo (11), pigs (4), and horses (3). Among the wild animals, mongooses (37) had the highest number of cases followed by civet (12) and other animals (Table 6). The mean annual number of mongooses confirmed positive was 3.1. The numbers of rabies positive mongoose samples were highest in 2001 (7) and 2002 (10). In other years number decreased to 0–3 samples (Figure 2).

**Table 6:** The total number of rabies positive samples in different animals during 12 year period.

Animals	Number of rabies positive samples	Category
Mongoose	37	
Civet	12	
Squirrel	9	
Jackal	8	
Rabbit	5	
Monkey	5	
Bandicoot	5	Wildlife
Wild cat	3	
Rock squirrel	3	
Deer	1	
Cow	110	Livestock
Horse	3	
Pig	4	
Buffalo	11	
Goat	26	

**Figure 3:**
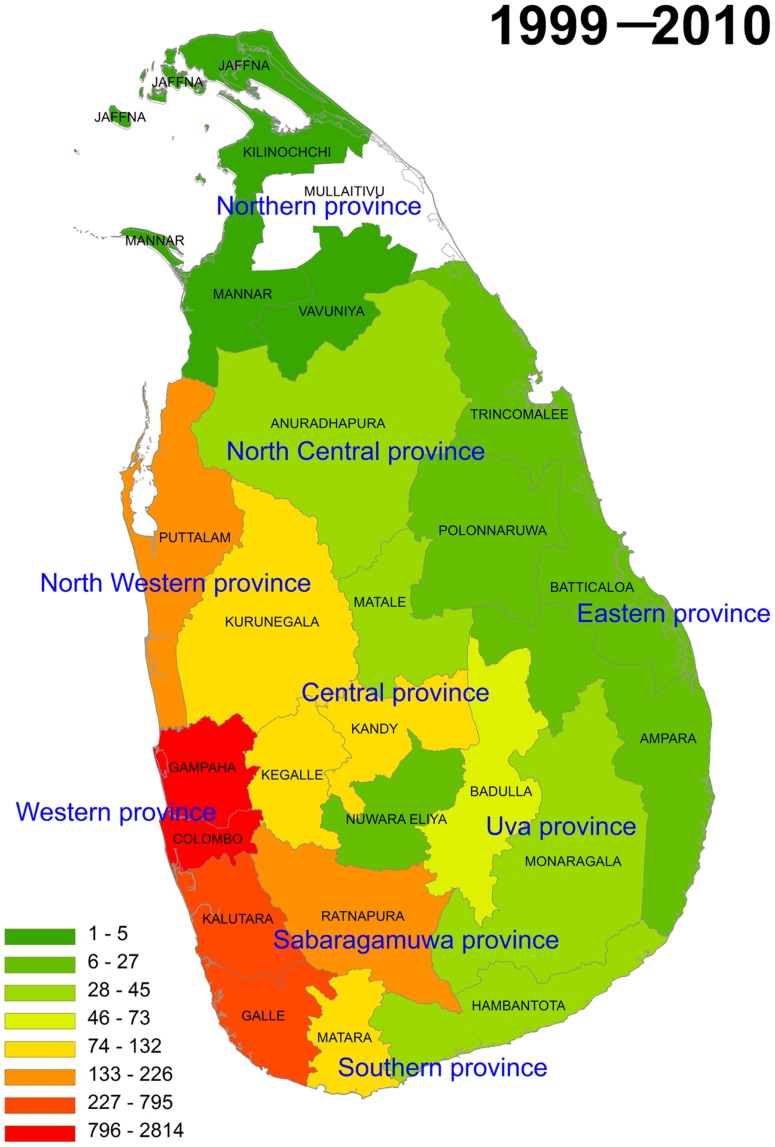
The cumulative number of animal rabies cases from 1999 to 2010 is plotted on the map. Rabies in Sri Lanka is highly prevalent in the districts of Western and Southern provinces. From 2004, rabies was detected in more areas from the eastern part of the country, and from 2007, rabies was detected in the northern district Jaffna. White indicates that no sample was received from this district.

**Figure 4:**
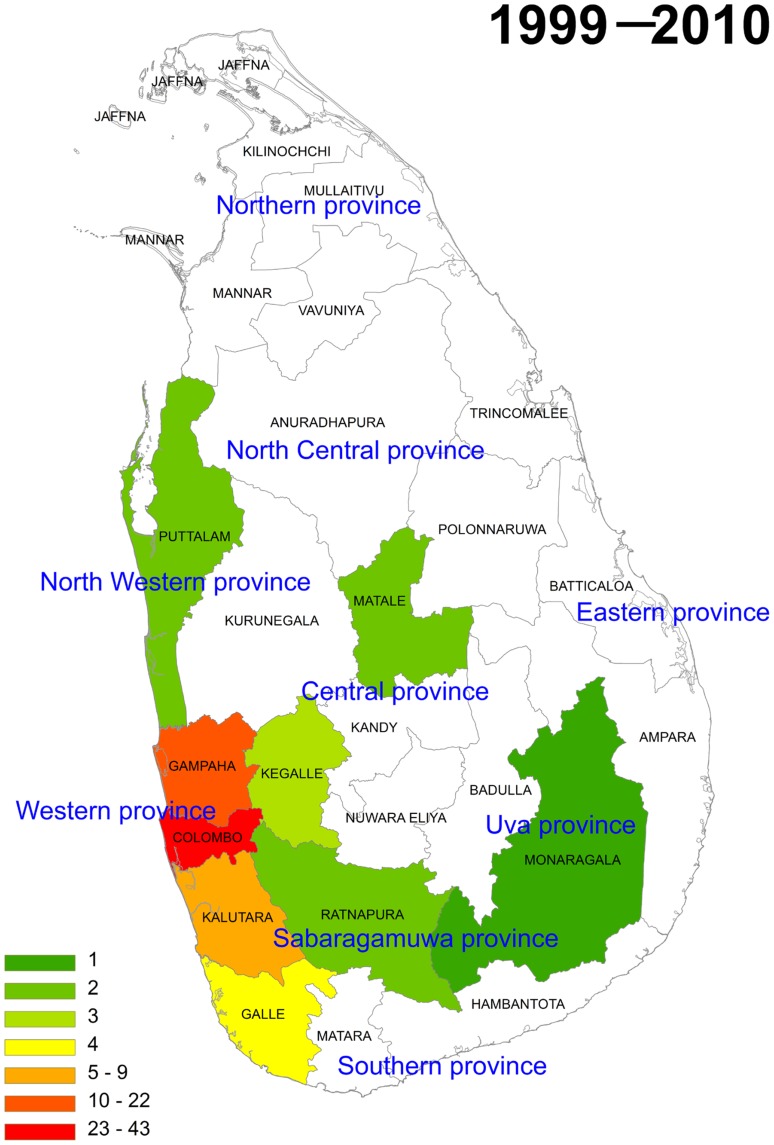
The cumulative number of wild animal rabies cases from 1999 to 2010 is plotted on the map. Wild animal rabies cases were mainly in the districts of Western, Southern, and Central provinces. No wild animal rabies cases were detected in other provinces possibly because fewer samples were received by the laboratory. White indicates that no sample was received from those districts.

The distribution of total animal and wild animal rabies cases from 1999 to 2010 was plotted by district on maps (Figure 3 and 4). There was a yearly fluctuation in the number of rabies cases when all animal submission were grouped together. From 2004, more cases of rabies were confirmed in the areas from the eastern part of the country. From 2007, rabies was confirmed in the northern district of Jaffna. Time-line mapping (Figure S1 and S2) and cumulative mapping (Figure 3 and 4) with the data available at the MRI showed that rabies was in high prevalence in the districts of Western and Southern provinces. By contrast, although there was a yearly fluctuation of the number of confirmed wild animal rabies cases, wild animal rabies was confirmed more frequently in the districts of Western, Southern, and Central provinces. No wild animal rabies cases were confirmed from other provinces.

### Human rabies

**Figure 5:**
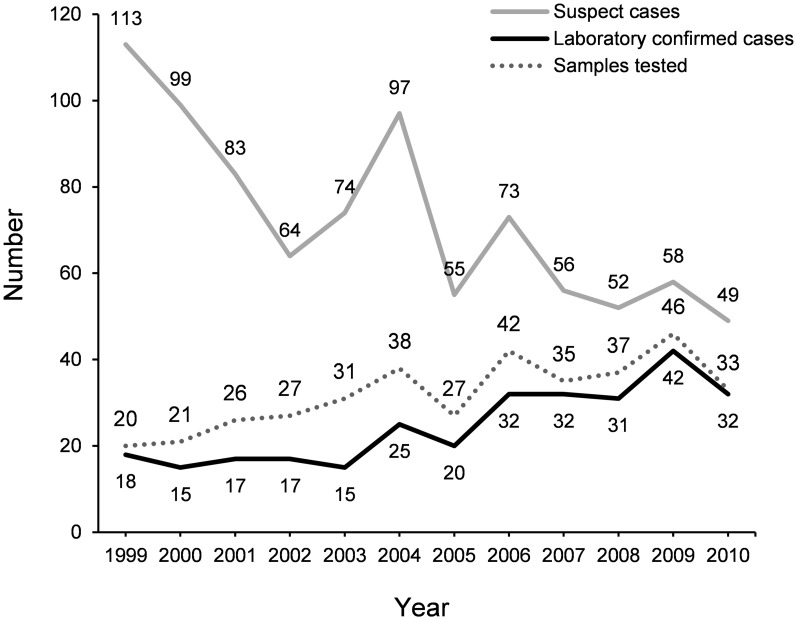
The number of submitted samples to confirm rabies and the number of laboratory confirmed human rabies specimens showing an increasing trend from 1999 to 2010 (dotted line and black line). There was an overall decreasing trend for human deaths from suspect rabies during the same period (gray line).

The number of laboratory-confirmed human rabies specimens increased from 18 in 1999 to 32 in 2010 (Figure 5). The lowest number of confirmed human rabies cases was in the years 2000 and 2003 when 15 human cases were detected. The highest number of human rabies cases (42 cases) was confirmed in 2009. Each year, an average of 24.7 (15–42) human samples was confirmed positive for rabies. The number of samples received increased from 20 in 1999 to 33 in 2010. The lowest number submitted in 1999 and the highest number of received samples (46 samples) in 2009. From 1999 to 2003 an average of 25 samples per year were received in the MRI, from 2004 to 2010 the average number increased to 36.8 samples per year. According to the Ministry of Health there is a trend toward an overall decrease in the number of human deaths from suspect rabies from 113 in 1999 to 49 in 2010. From these data, the mean number of human deaths from suspect rabies per year was calculated as 72.7 (49–113). The Sri Lankan government expenses on PEP for the year 2005, 2006, 2007, 2008, 2009, and 2010 were 302.9. 204.2, 197.8, 286.9, 378.6, and 351.4 million Sri Lankan Rupees, respectively. The Pearson correlation coefficient value of R was −0.5432, indicating that there is a moderate negative correlation between the annual number of human deaths from suspect rabies and annual expenditure of Sri Lankan government on rabies PEP.

Samples were received from 18 of 25 districts in nine provinces of Sri Lanka. When classified by district, rabies-positive samples were mainly submitted from Colombo, Gampaha, Galle and Kalutara (Table 7). When classified by province, the numbers of positive samples(percentage of the total positive samples from all provinces)/the numbers of submitted samples during the 12 years were submitted in the following order: 86 (29.0%)/118 from Western province, 60 (20.3%)/85 from Southern province, 37 (12.5%)/47 from North Western province, 27 (9.1%)/33 from Uva province, 22 (7.4%)/23 from Sabaragamuwa province, 22 (7.4%)/29 from Central province, 20 (6.8%)/24 from North Central province, 15 (5.1%)/16 from Eastern province, and 7 (2.4%)/8 from Northern province.

**Table 7:** The annual number of human rabies cases in all provinces and their respective districts of Sri Lanka.

Year	Central			Western			Southern			Eastern			Northern					NW		NC		Sabaragamuwa		Uva		Total
	MT	KY	NW	CO	GQ	KT	GL	MH	HB	TC	APR	BC	JA	MB	VA	MP	KL	PX	KG	AD	PR	RN	KE	MJ	BD	
1999	0	2	0	5	2	0	6	0	0	0	0	0	0	0	0	0	0	0	0	1	0	0	0	0	2	18
2000	0	2	0	0	5	0	0	0	0	0	1	0	0	0	0	0	0	0	0	0	0	6	0	0	1	15
20001	2	1	0	2	1	1	3	2	0	0	0	0	0	0	0	0	0	0	1	0	0	2	0	0	2	17
2002	0	1	0	1	5	0	3	1	0	0	0	0	0	0	0	0	0	1	2	0	0	1	1	0	1	17
2003	0	0	0	0	3	0	3	1	0	0	0	0	0	0	0	0	0	2	2	0	0	0	0	3	1	15
2004	0	0	0	4	1	4	7	6	0	0	0	0	0	0	0	0	0	0	1	0	0	0	1	1	0	25
2005	0	0	1	4	2	2	1	0	1	2	0	0	0	0	1	0	0	0	0	1	0	2	1	0	2	20
2006	1	2	0	5	0	2	6	2	0	0	0	0	0	1	0	0	0	4	0	3	0	1	1	0	4	32
2007	1	2	0	4	0	3	2	3	0	0	0	0	0	0	1	0	0	0	8	3	0	2	0	1	2	32
2008	0	3	0	3	2	2	4	1	0	1	0	1	0	0	1	0	0	2	7	2	1	0	0	1	0	31
2009	1	1	0	9	3	3	5	1	0	1	0	3	0	0	0	0	0	2	2	5	0	1	1	4	0	42
2010	1	1	0	5	2	1	0	1	1	0	0	6	1	0	1	0	1	1	2	4	0	2	0	2	0	32
Total	6	15	1	42	26	18	40	18	2	4	1	10	1	1	4	0	1	12	25	19	1	17	5	12	15	296

Abbreviation of provinces and districts are shown below: North Western province; NW, North Central province; NC, Ampara; APR, Anuradhapura; AD, Badulla; BD, Batticaloa; BC, Colombo; CO, Galle; GL, Gampaha; GQ, Hambantota; HB, Jaffna; JA, Kalutara; KT, Kandy; KY, Kegalle; KE, Kilinochchi; KL, Kurunegala; KG, Mannar; MB, Matale; MT, Matara; MH, Moneragala; MJ, Mullaitivu; MP, Nuwara Eliya; NW, Polonnaruwa; PR, Puttalam; PX, Ratnapura; RN, Trincomalee; TC, and Vavuniya; VA.

The mean yearly numbers (range) of positive samples were confirmed from provinces in the following order: 7.2 (3–15) from Western province, 5.0 (0–13) from Southern province, 3.1 (0–9) from North Western province, 2.2 (1–4) from Uva province, 1.8 (0–6) from Sabaragamuwa province, 1.8 (0–3) from Central province, 1.7 (0–5) from North Central province, 1.2 (0–6) from Eastern province, and 0.6 (0–3) from Northern province.

## Discussion

The MRI is the national reference center for rabies in Sri Lanka and therefore reflects the rabies situation throughout the country. We should be cautious when interpreting the low number of rabies-positive specimens received from Eastern and Northern Provinces because the conflict situation hampered the sampling and transport of suspected specimens to the MRI. The situation returned to normal in 2003 in Eastern Province, in 2007 in the Jaffna district, and in 2009 in the entire Northern Province. In 2005 and 2010, the lowest percentage of rabies was detected. The tsunami on December 26, 2004 may have affected the rabies situation in 2005. The conflict situation peaked and floods affected the country in 2009 and 2010, respectively. These events may have interrupted sample collection and transport. It has been documented that intense conflicts may increase the incidence of rabies [11]. With relatively long incubation period of rabies, the time taken for a system to become smooth for sample collection and testing, and the nature of rabies surveillance it may take several years before the increase is recognized.

Samples not received from different areas of the country are a concern when trying to document the prevalence of rabies in wild animals. Greater awareness among the public is needed for the surveillance of rabies in wild animals from these provinces. The animal samples submitted to the MRI were submitted mainly by the general public, and forest rangers and veterinarians also contributed some animal samples. The limitation of this sample collection system is that about 40% of the samples were found to be rabies negative indicating many of the suspect rabies animals did not have rabies or the transportation of samples were not adequate enough and as a result the samples were FAT negative. Further limitation is that the general people are at risk to contract rabies when they catch a suspected rabies animal. These situations can be avoided if a professional sample collection system can be established. Although bats are commonly seen in Sri Lanka, only one sample from a bat was submitted for the first time in 2010. Because no bat rabies has been reported and bats are a protected species in Sri Lanka, bat samples would not have been submitted for screening. However, this area needs to be explored.

Similar to the patterns in other rabies-endemic countries of Asia and Africa, dogs are the main reservoir species of rabies in Sri Lanka. Rabies epidemiology is highly responsive to human- and dog-population densities as well as to the cultural and socioeconomic environments [12]. In Sri Lanka the estimated dog population density is 108 dogs/km^2^ [13], which is much higher than the threshold density of 4.5 dogs/km^2^ necessary for the persistence of rabies [14].

The incidence of rabies in livestock is an important factor for estimating the economic impact of the disease [15]. However, rabies in livestock has not been studied thoroughly, and the impact of rabies has been reported in a limited number of papers [16], [17]. There are an estimated 1.5 million cattle, 0.3 million buffalo and 0.08 million pigs in Sri Lanka, and livestock contributes 1.2% of this country's gross domestic product (http://www.livestock.gov.lk/site/en/statistics). We are of the opinion that the numbers of submitted samples found in the present study do not represent the true prevalence of rabies in Sri Lankan livestock. There is no surveillance system in Sri Lanka for livestock rabies and, as mentioned above, most animal samples for rabies testing are submitted by the public. It may be difficult to submit samples of livestock because of the large head of these animals, which may increase the cost of transportation and maintaining the cold chain. In addition, there is no compensation scheme in Sri Lanka for the loss of livestock, and farmers are not encouraged to submit livestock samples to exclude rabies when the cause of death is unknown. Therefore, we recommend the establishment of a surveillance system for livestock in Sri Lanka. This will help quantify the economic loss from rabid livestock, and would help prevent the entry of the rabid animal into the food chain and thereby avoid human contact during carcass disposal.

There appears to be no temporal relationship between rabies cases in livestock when compared to the annual number of rabies in dogs and other animals, suggesting that there may be a complex relationship that cannot be explained by our data alone. As found for other rabid animals most of the rabid livestock samples were from the Western and Southern provinces (data not shown), although this still leaves the geographical relationship unanalyzed. Patterns of infection in livestock depend upon the infection dynamics of the reservoir species within a particular area and opportunities for contact between livestock and reservoir species [18]. It is possible that livestock rabies in Sri Lanka is transmitted mainly by dogs, as reported from Bhutan and the Serengeti region of Tanzania [19], [20]. Other than dog and cat samples, the highest number of samples was obtained from wild animals. However, we may assume that in all cases involving livestock and wild animals, rabies may not be transmitted by dogs only, and there might be sylvatic transmission of rabies. Other factors can affect transmission, such as the frequency of contact between dogs and livestock, and these factors are affected by the number of animals, availability of food, infections, climate, and human activities.

Mongooses were the main victims of rabies among wild animals. In the Southern province of Sri Lanka, rabies has been detected by FAT in 22% of the free-roaming mongooses found in gardens, showing that the mongoose is a reservoir species of rabies in the wild ecosystem of Sri Lanka [21]. Whether the rabies virus detected in mongooses is a separate variant from the dog lineage, as found in South Africa [22], should be confirmed in future studies since in this study genetic typing of rabies viruses were not performed. In this retrospective analysis, rabies was not detected in any samples of civets. We note that rabies is difficult to identify with FAT if the sample is not fresh [10], [23]. In our rabies laboratory, FAT is not performed when decomposed tissues are identified. However, it is a possible that some of the samples from remote places, were not fresh enough to detect rabies using FAT and that visual examination was not sufficient to identify decomposed samples. Therefore to improve the surveillance of rabies in Sri Lanka introduction of molecular techniques might be useful, that may also help to develop a detailed map of rabies virus variants throughout Sri Lanka and will enable a more successful rabies elimination program in the future.

We note also that the reported civet with sylvatic rabies virus was recorded as a wildcat in the laboratory registry [6], indicating that the persons who received the animal head may have had difficulty identifying wild animals. Having a mechanism to identify wild animals by using photographs or to train the staff receiving samples would help prevent misclassification. Three rabid wildcats were detected in 2009, one of which was available for complete genetic analysis and was reported as the first sylvatic rabies case in a golden palm civet in Asia [6].

Rabies surveillance in wildlife is extremely difficult to undertake and therefore it is currently unknown in Sri Lanka. Possibly modest inferences can be made from the present study about the rabies status of wild animals in Sri Lanka because only wild animals with a human interaction were tested in this study and many potentially rabies wild animals are never discovered or tested. Therefore a major study is necessary to describe rabies in the Sri Lankan wildlife populations.

Human rabies is a notifiable disease in Sri Lanka. Following the issue of an official circular (General Circular No. 01-22/2004) by the Sri Lankan Ministry of Health, indicating that an autopsy should be performed on all clinically diagnosed human rabies cases. Although there has been a yearly variation the number of human samples received in the MRI has increased after the official circular. This practice should be followed by other countries of this region, where people are reluctant to perform autopsy for sociocultural reasons. A main finding in our study is the increase in the number of laboratory confirmed human rabies cases and the increase in the number of samples submitted for laboratory confirmation of rabies. Data from the Ministry of Health showed that the number of suspect human rabies cases has been decreasing gradually in Sri Lanka. All of these suspect human rabies cases were not confirmed by laboratory test. The laboratory confirmed and suspect rabies cases are not reported separately, otherwise it would be better for understanding the epidemiology of rabies. This study analyzed data collected over 12 years and our estimated number of human deaths from suspect rabies was higher than other estimates of <60 deaths per year [24]. Except for the 73 deaths in 2006, from 2005, the number of human deaths from suspect rabies in Sri Lanka was below 60.

The national rabies control program was initiated in Sri Lanka in the 1980s with the introduction of dog vaccination, elimination of stray dogs, postexposure prophylaxis (PEP) for suspected animal bite victims, and other related issues [13]. Since 2005, dog elimination has been replaced by dog birth control having a national coverage of 2.3% in 2006. [25]. The national coverage of dog vaccination was 49.3% in 2007. This coverage is inadequate to break the natural transmission cycle of the rabies virus [25]. Dog birth control rates of>80% and dog vaccination rates of>70% are required for an effective campaign. To achieve these goals, a strong political will, mobilization of budget, mobilization of experts, supports from religious leaders, and public awareness are necessary. At present people are aware of the anti-rabies protective measures that should be taken following an animal bite and Sri Lanka has experienced modest success in its rabies control program [3]. The dog population of Sri Lanka is more than 2.5 million [26]. Full-scale control programs cannot be implemented in many developing countries because of budget and technical constraints. Sri Lanka is not an exception and should begin programs in Western and Southern provinces where the highest number of rabies were reported. In this study we found that the number of rabies positive samples increases with the number of population or population density of the area, and Western and Southern provinces have one of the highest population and population density in Sri Lanka. However sampling might be affected by human population centers, distance to rabies laboratories, economics of submitting animals and other factors which are unavoidable in such kind of studies performed in any parts of the world. In order to expand and improve surveillance, it would be helpful to establish rabies diagnostic laboratories in other provinces.

This study showed that, in Sri Lanka, the number of cases of dog rabies did not change significantly over the study period. The main reasons for the decrease in the number of human with suspect rabies in Sri Lanka is not in particular due to the efforts adopted for dog control; rather, it reflects the government's effort to provide easy access to PEP free of charge to animal bite victims in government hospitals, and the public awareness of rabies created through education and the media [3]. The number of human rabies deaths and PEP correlate closely with the numbers of confirmed animal cases [27]. Collecting data on the number of people treated with PEP in all government hospitals was not possible therefore we used the government expenditure on rabies PEP as a method to estimate the number of doses of vaccine. A negative correlation between suspect human rabies cases in Sri Lanka and government expenditure on rabies PEP might supports our conclusion, however to confirm our results, further study is needed using the number of people treated with PEP at all government hospitals.

Natural rabies epidemics in southern and eastern Africa have demonstrated a cycle with a period of 3–6 years [27]. The cycle disappears when intensive vaccination programs in animals are implemented [27]. Some measures implemented in dogs may have caused the fluctuation in the incidence of rabies; we found no evidence of a periodicity of rabies occurrence in Sri Lanka.

### Conclusion

The number of cases of dog rabies has remained stable, indicating that the decrease in the number of human deaths from rabies reflects the government's efforts in implementing improved access to PEP to people exposed to suspected rabies infection. Public awareness of rabies might be another factor contributing to the decreasing trend in the number of human rabies. Fully integrated dog-control activities to cover the whole country would help decrease the incidence of dog rabies significantly in Sri Lanka. Establishment of regional rabies laboratories in other provinces will improve the surveillance of rabies throughout the country. Public awareness should be increased to understand the effects of rabies in livestock and wild animals. Greater cooperation from forest rangers and personnel dealing with wild animals is necessary to understand the overall picture of sylvatic rabies in Sri Lanka. It is of critical importance to perform genetic typing of samples submitted for rabies testing throughout Sri Lanka to find out whether multiple rabies virus variants are circulating within the country. This would be extremely valuable for describing the epidemiology, making PEP recommendations, and developing a comprehensive rabies elimination strategy for Sri Lanka and could serve as a model for other Asian countries.

## Supporting Information

Figure S1 The distribution of total animal rabies cases in different districts from 1999 to 2010.
(PDF)

Figure S2 The distribution of wild animal rabies cases in different districts from 1999 to 2010.
(PDF)
