# Supplementary figures and images for: Twelve Years of Rabies Surveillance in Sri Lanka, 1999–2010
Source: PLoS Negl Trop Dis. 2014 Oct 9;8(10):e3205. doi: 10.1371/journal.pntd.0003205 (PMC4191952; doi:10.1371/journal.pntd.0003205)

**Figure S1. The distribution of total animal rabies cases in different districts from 1999 to 2010.**

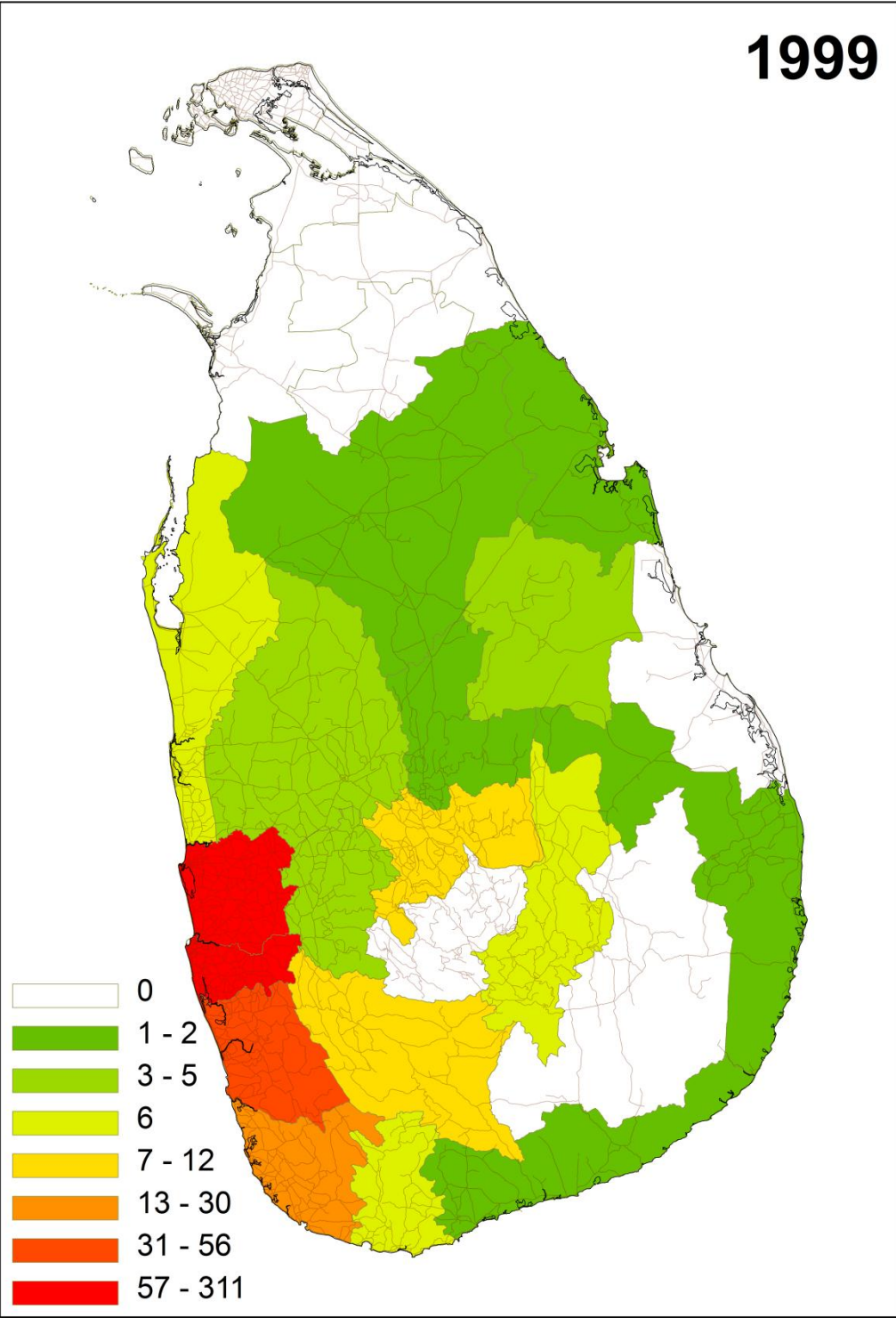

**2000**

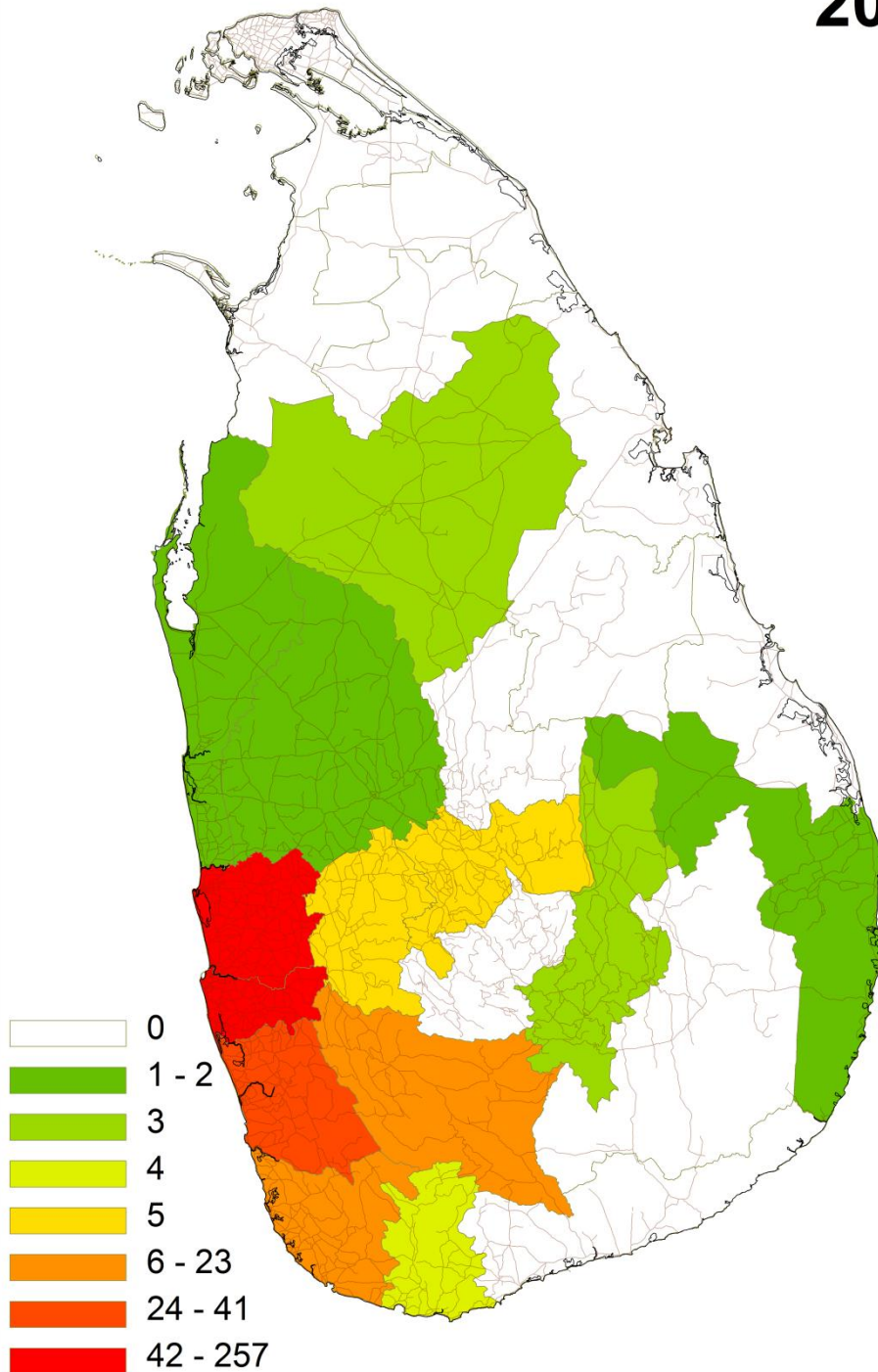

**2001**

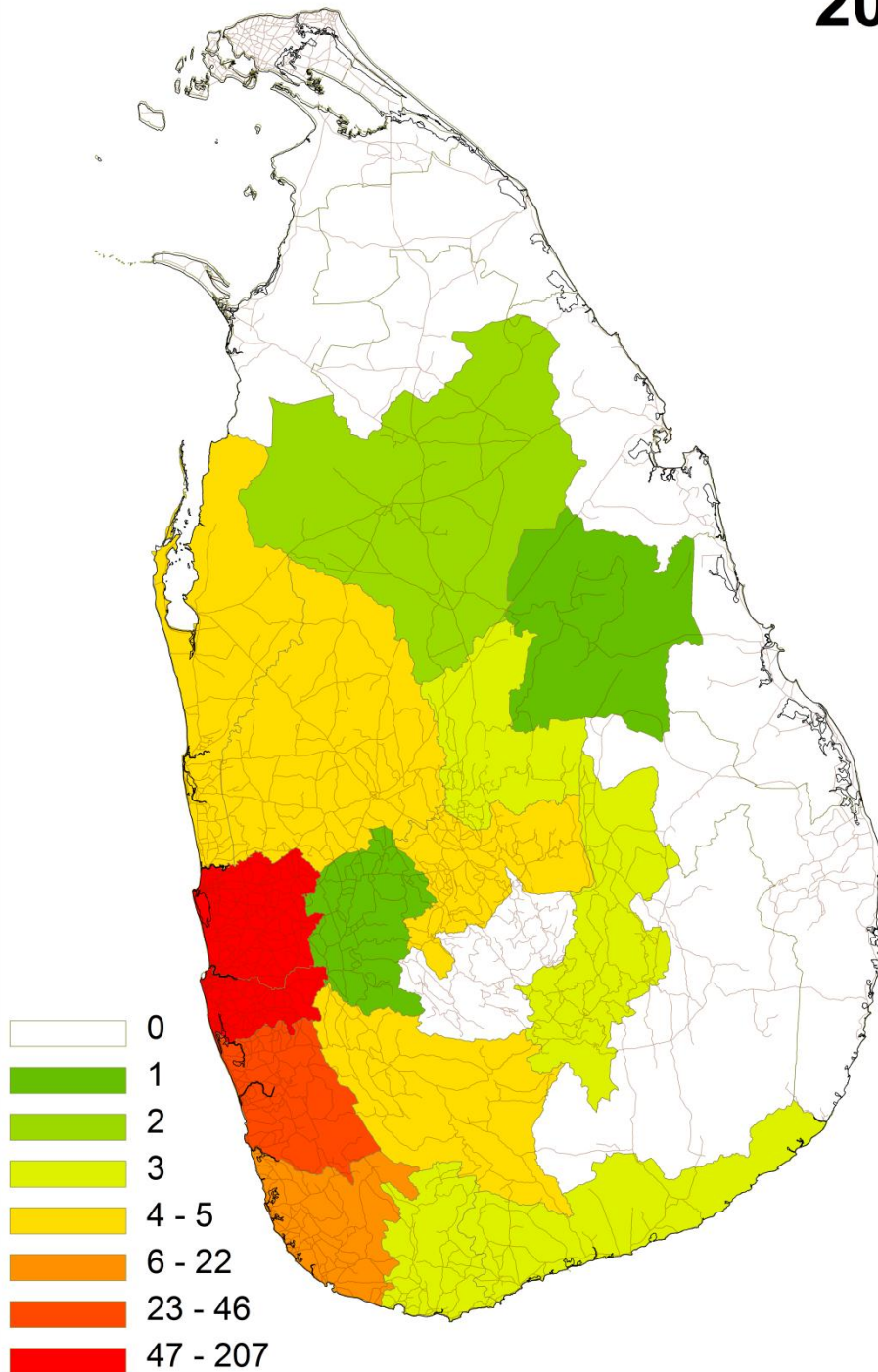

**2002**

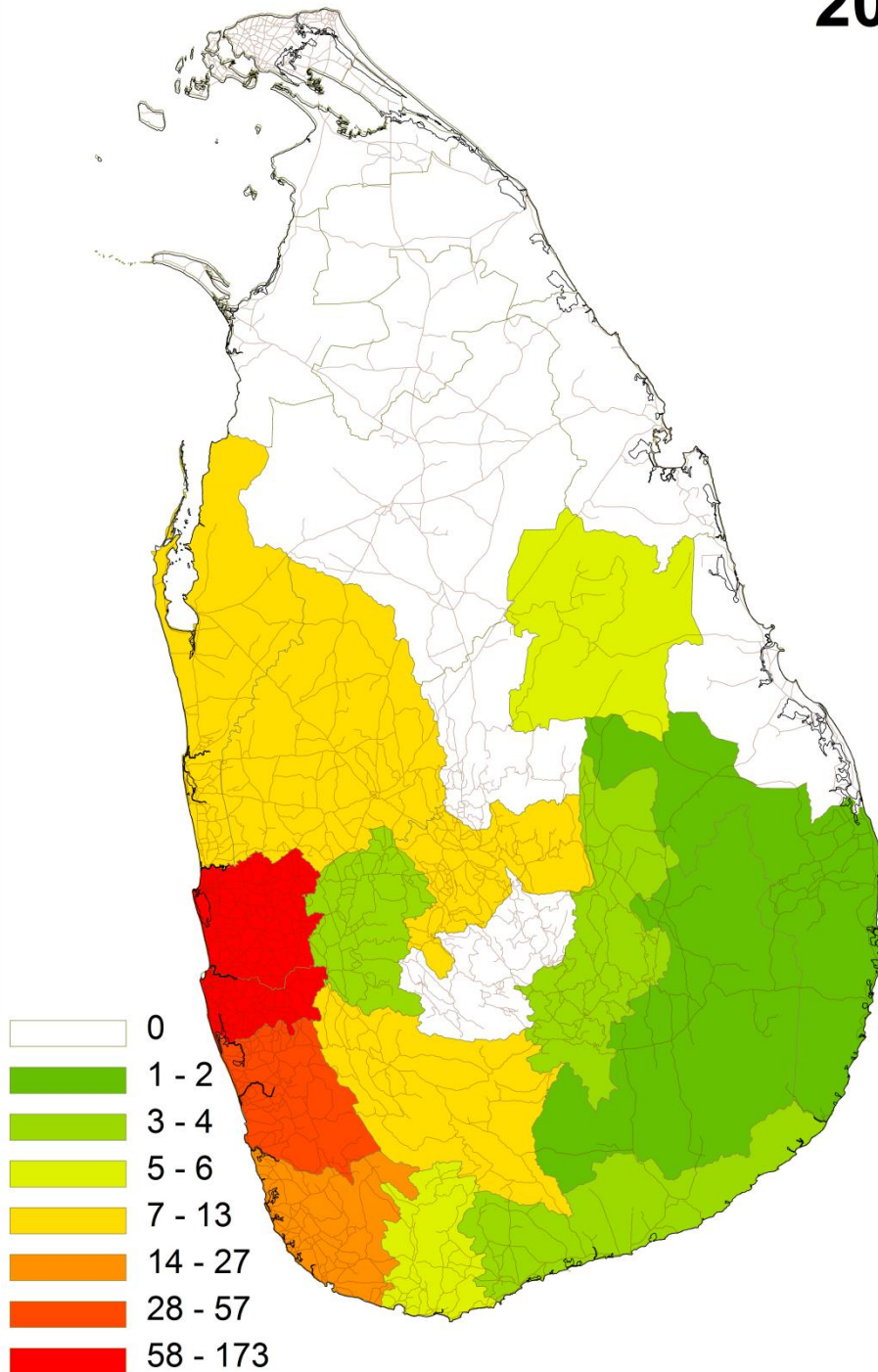

**2003**

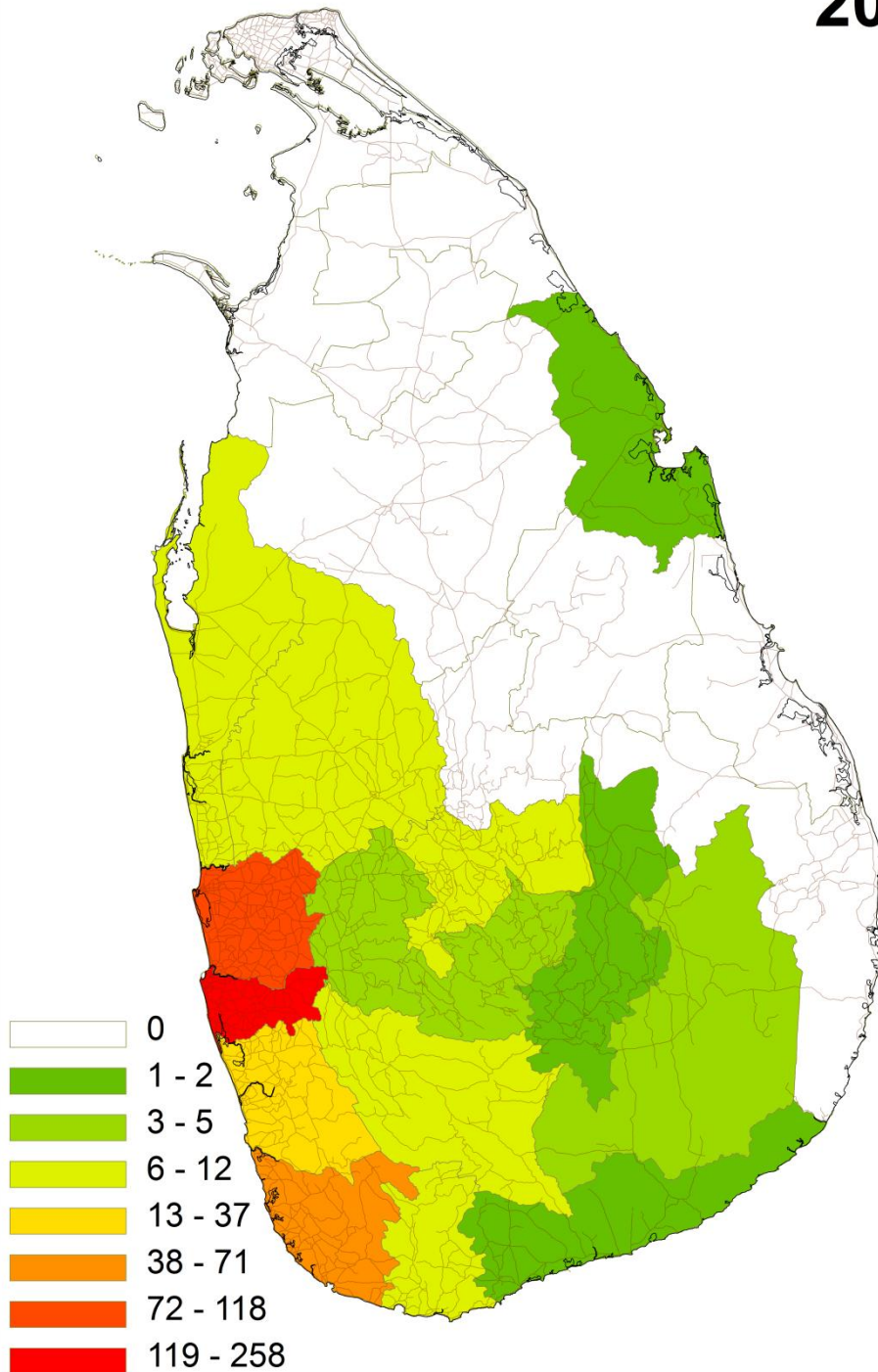

**2004**

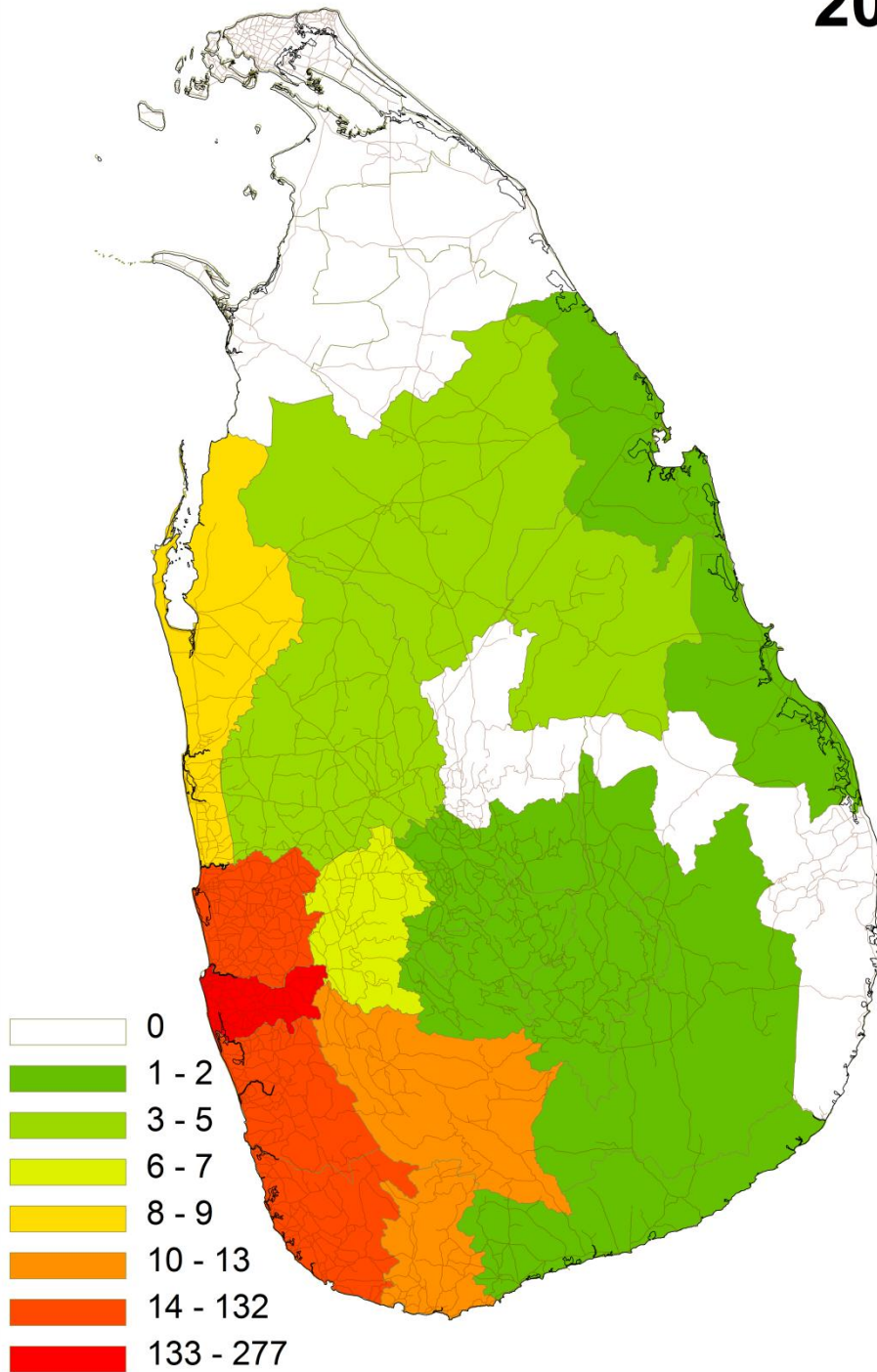

**2005**

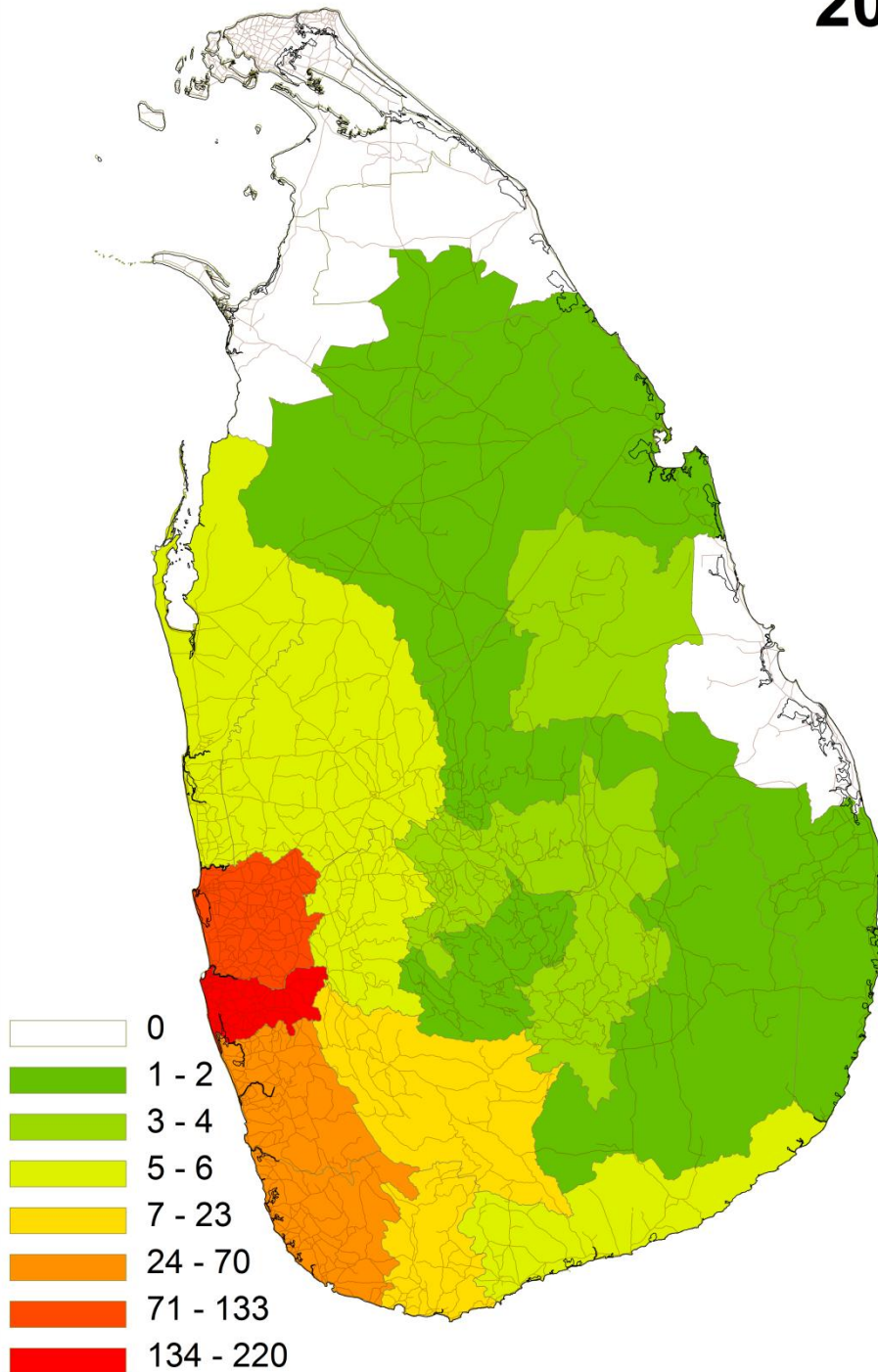

**2006**

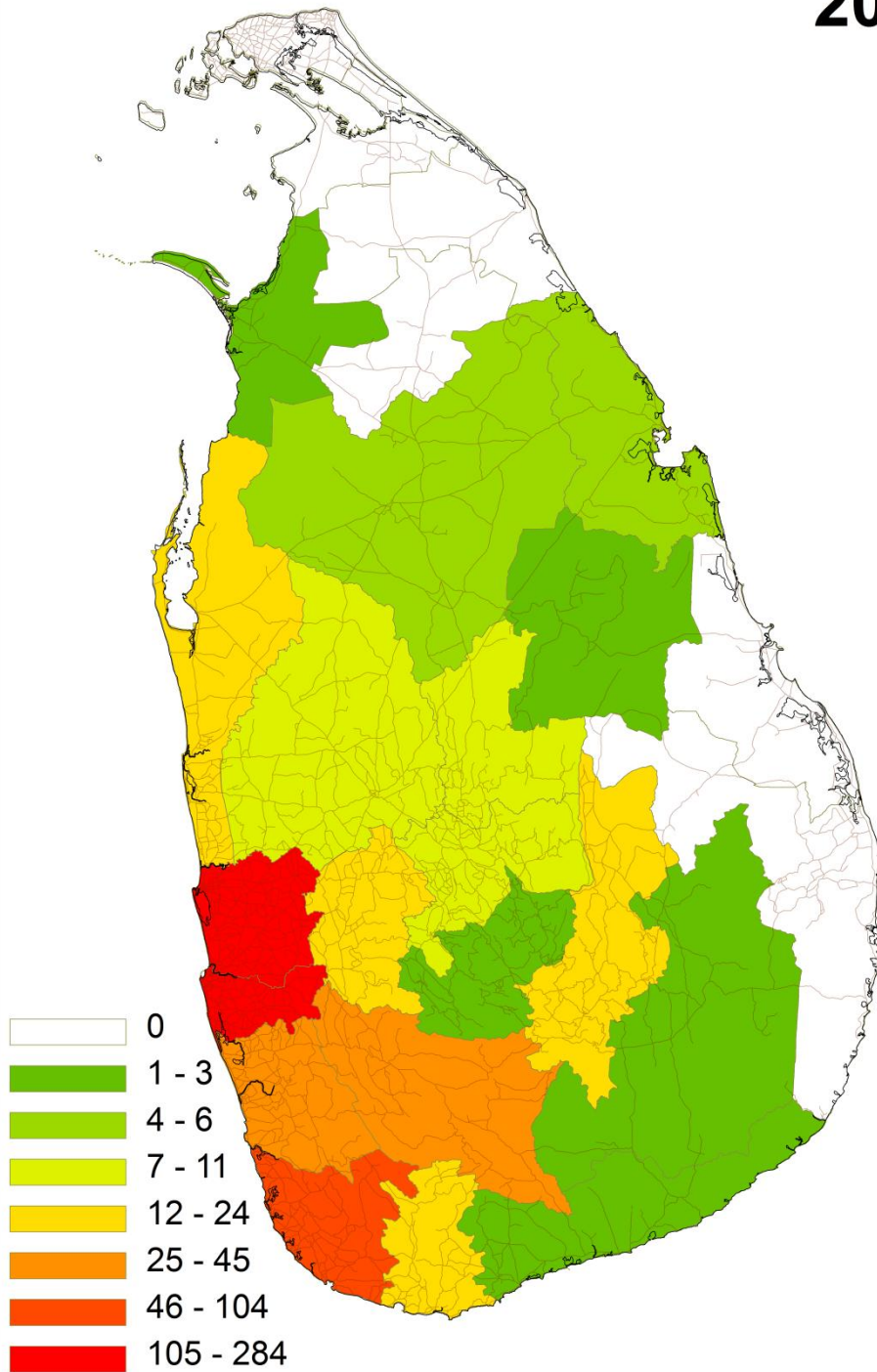

**2007**

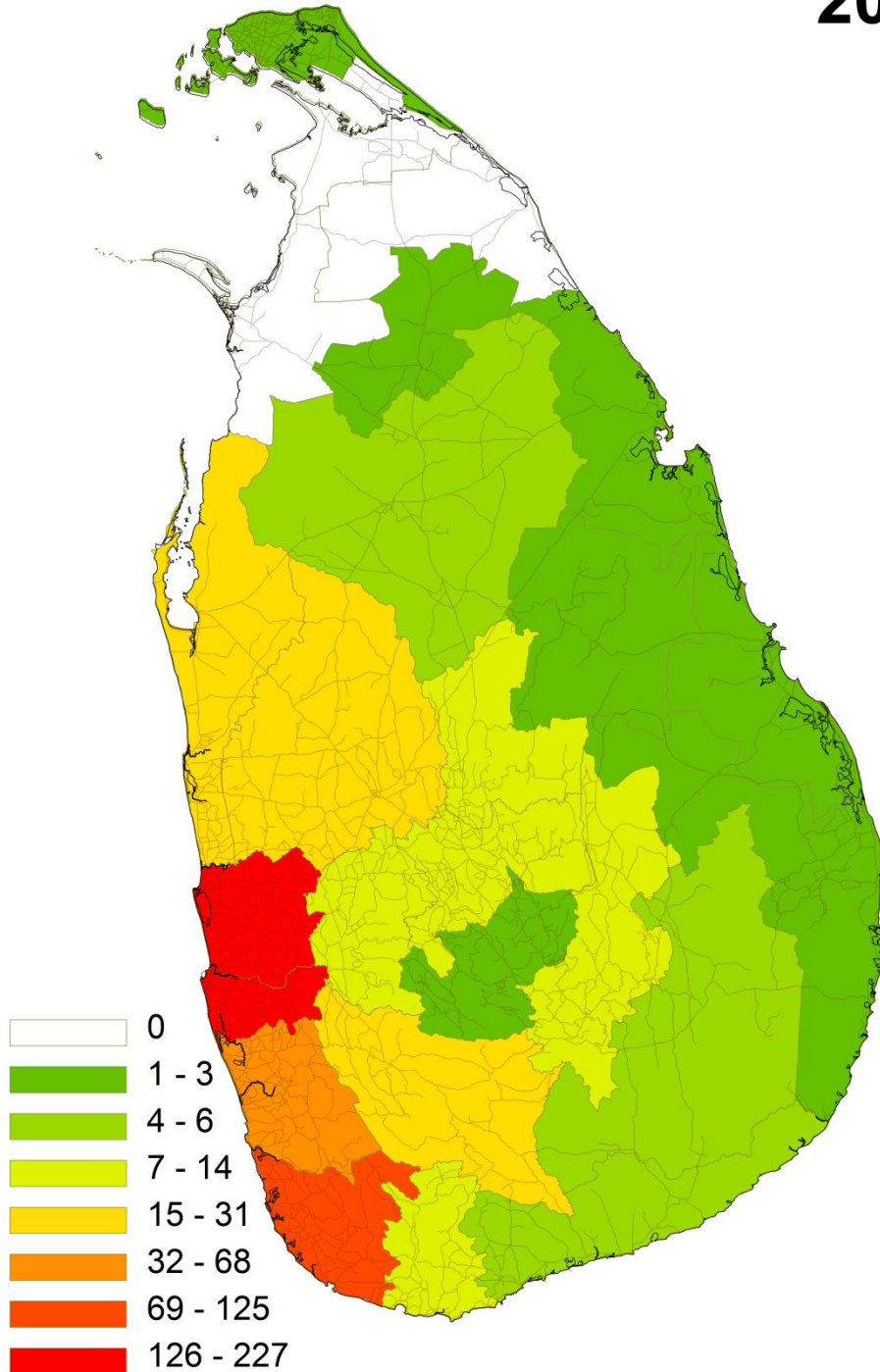

**2008**

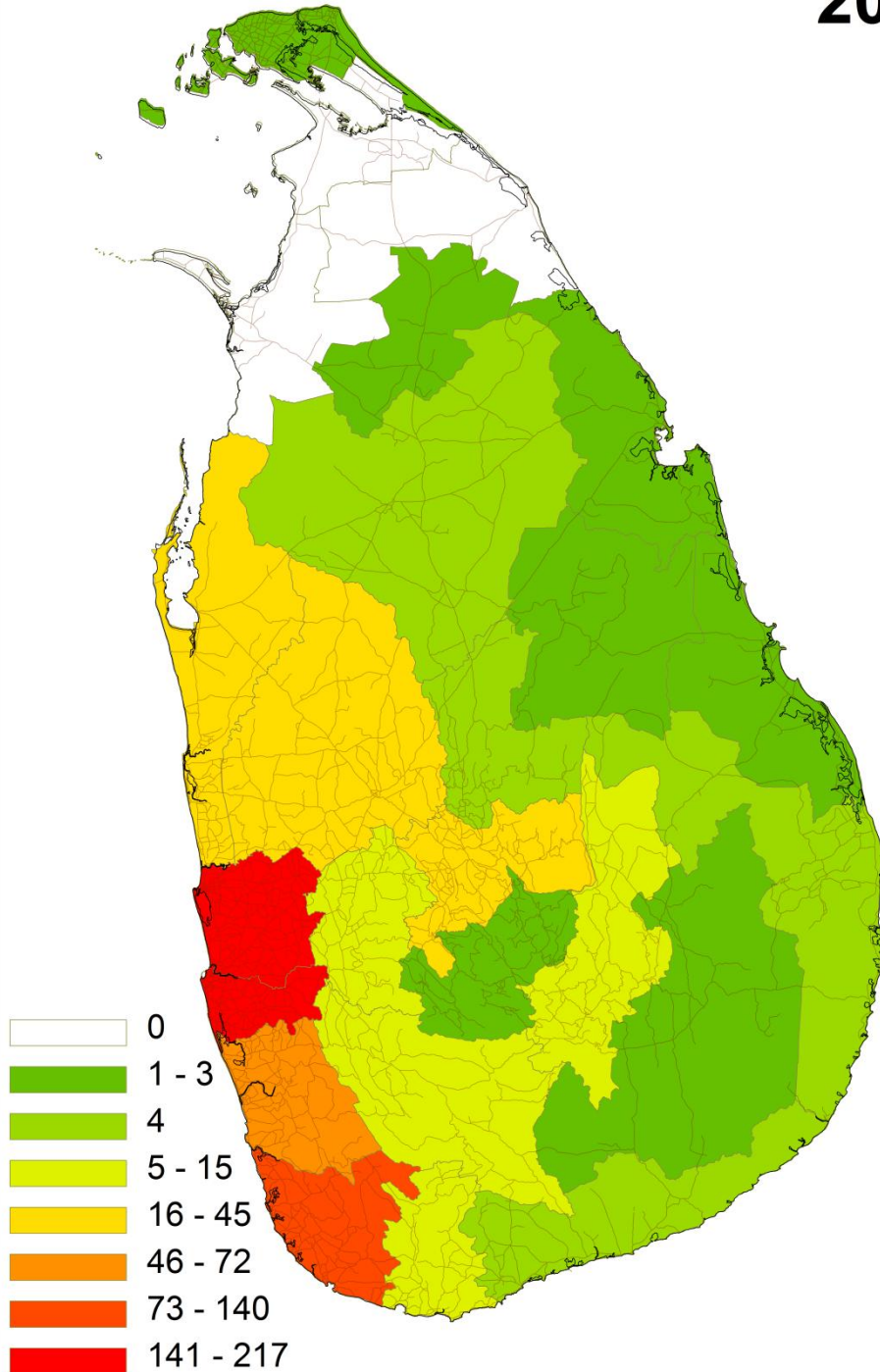

**2009**

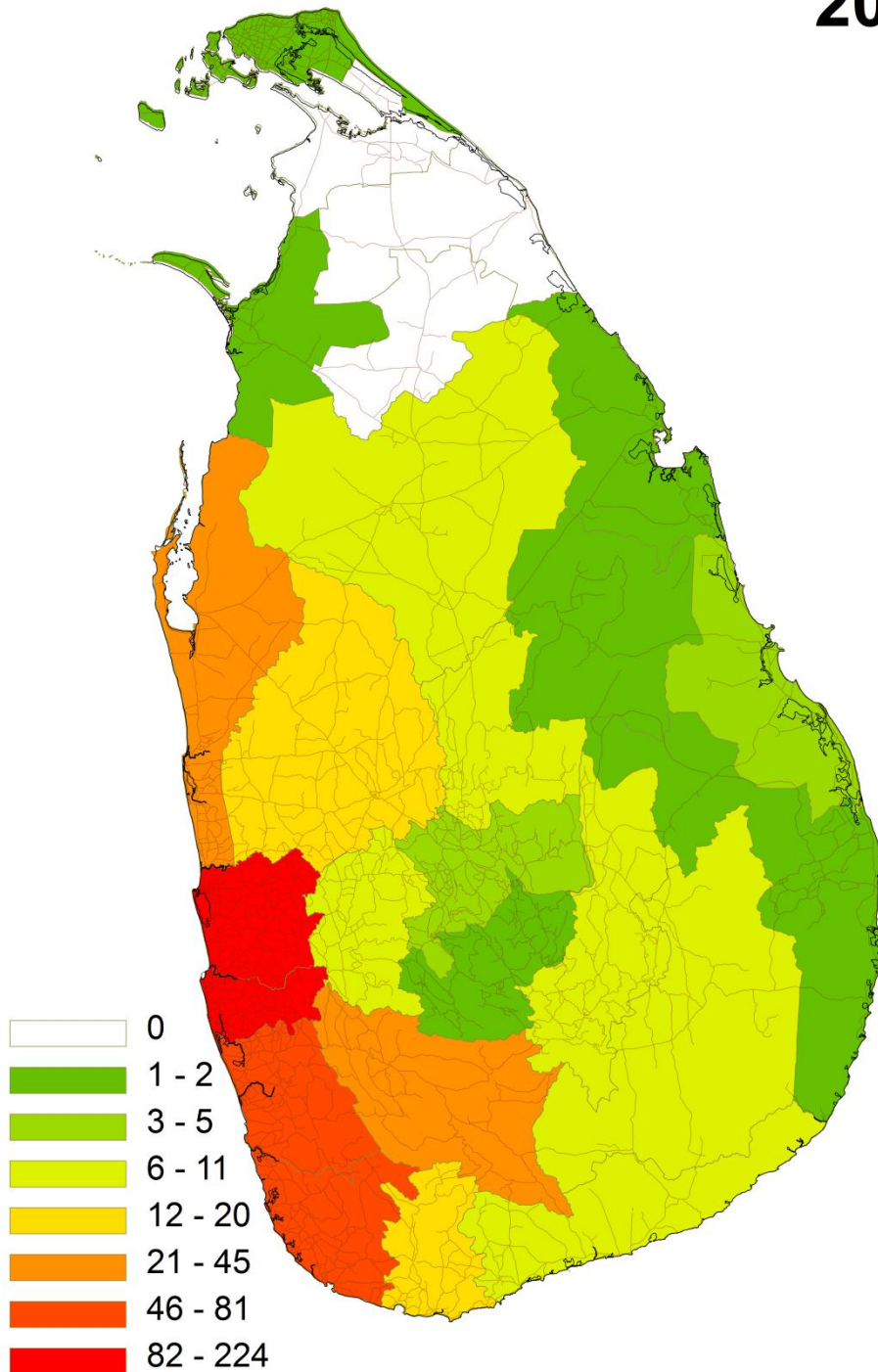

**2010**

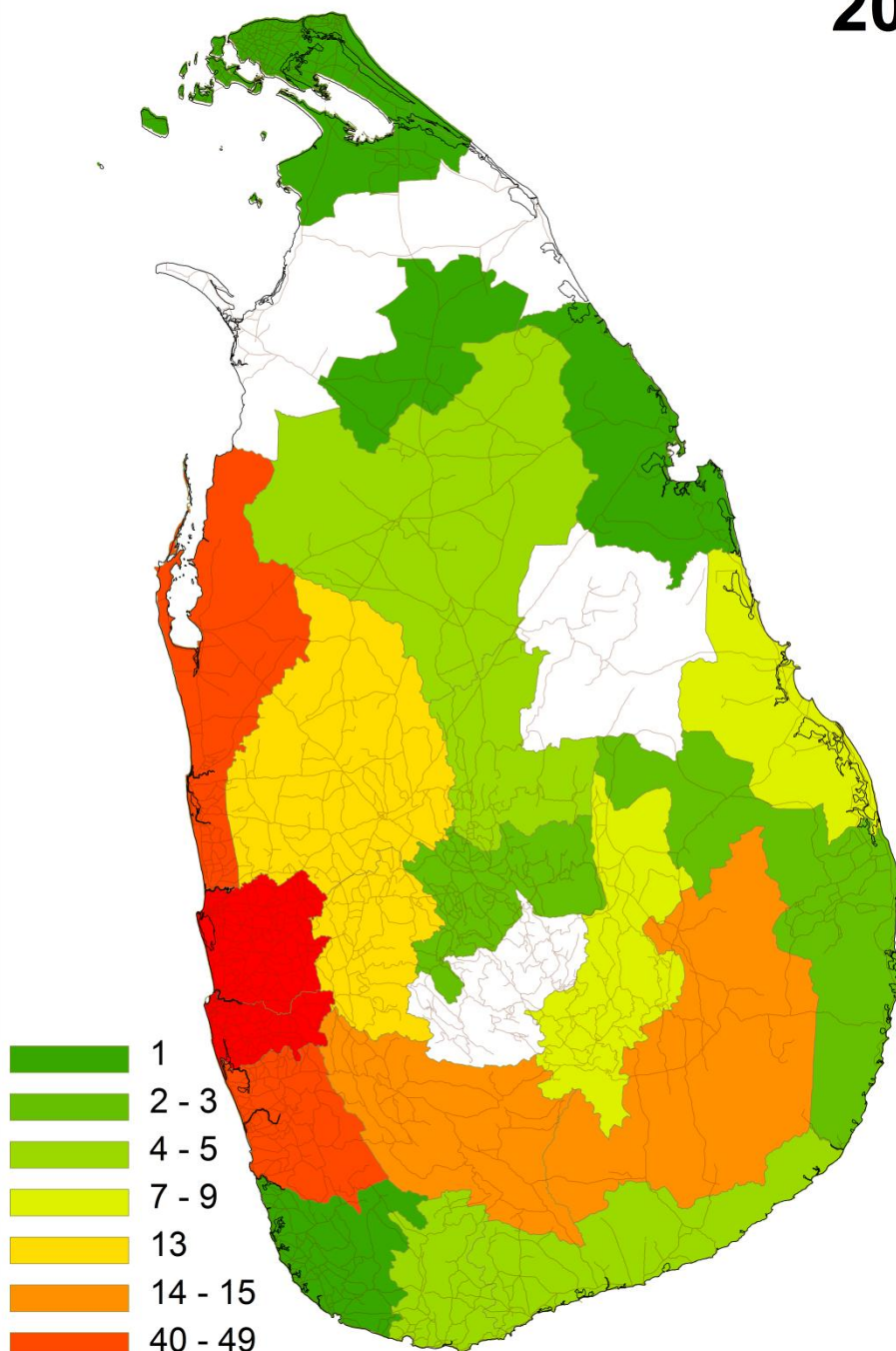

Supplement: Figure S1 — The distribution of total animal rabies cases in different districts from 1999 to 2010. (PDF) [file pntd.0003205.s001.pdf]

**Figure S2. The distribution of wild animal rabies cases in different districts from 1999 to 2010.**

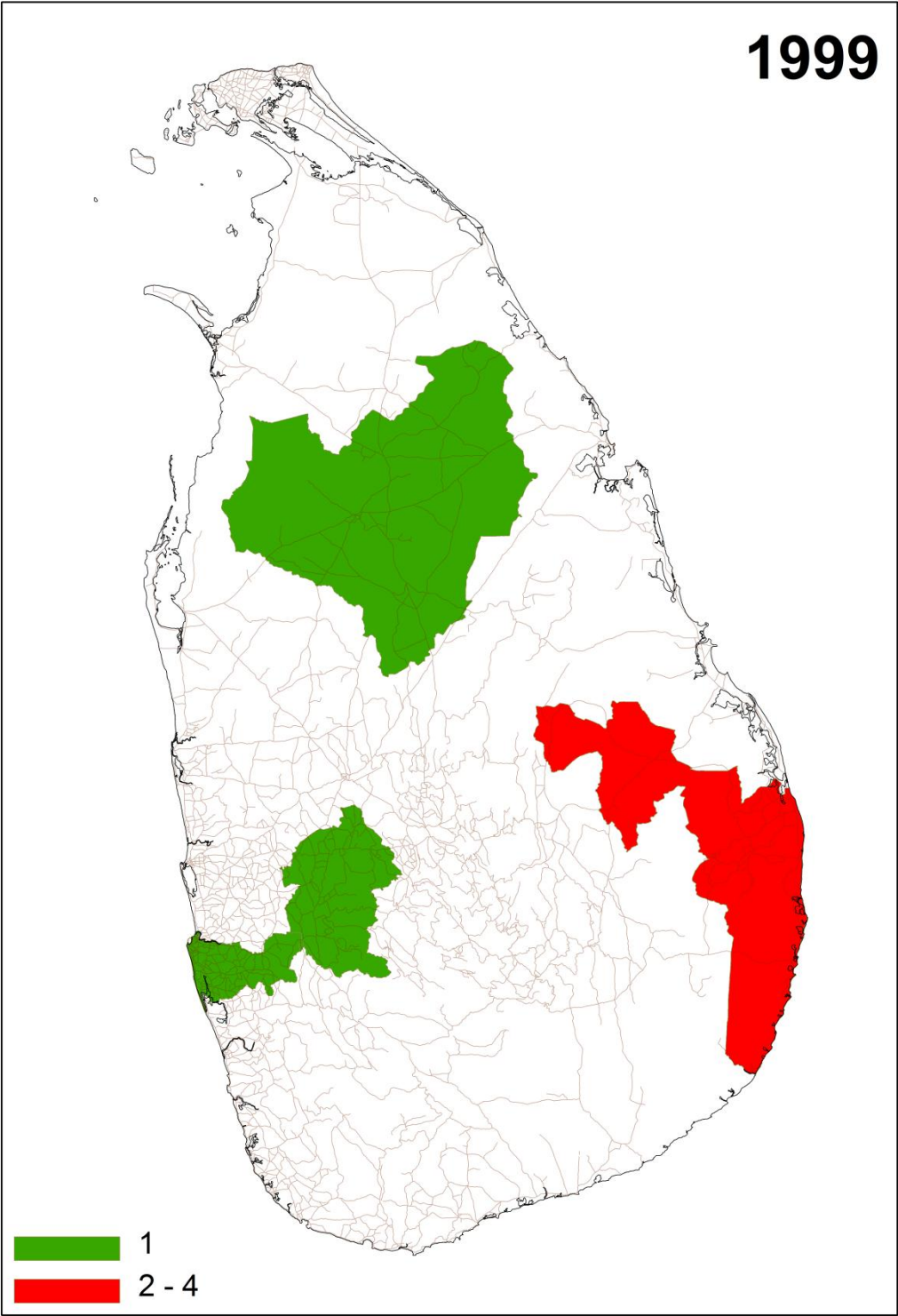

**2000**

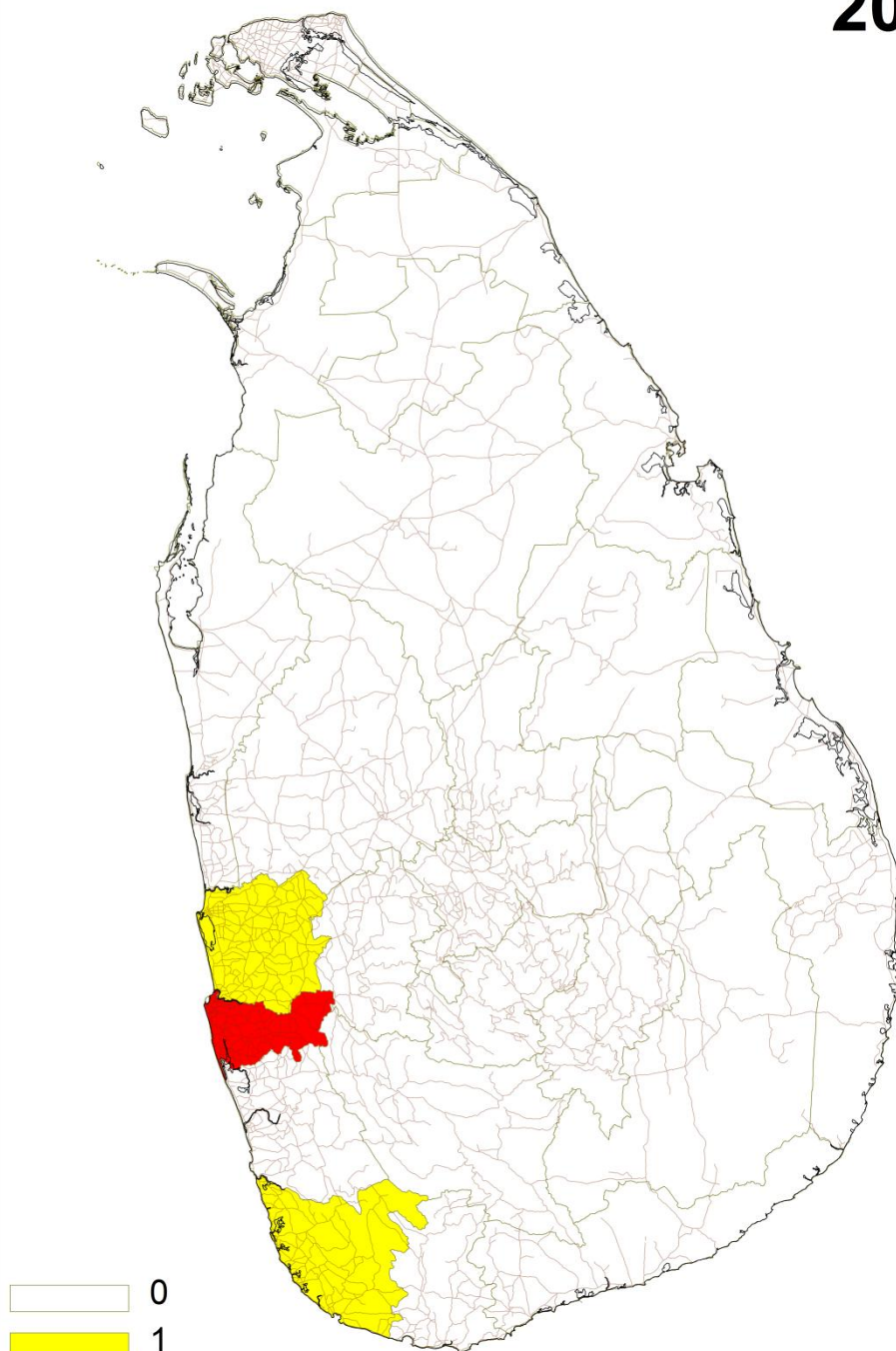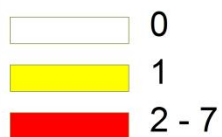

**2001**

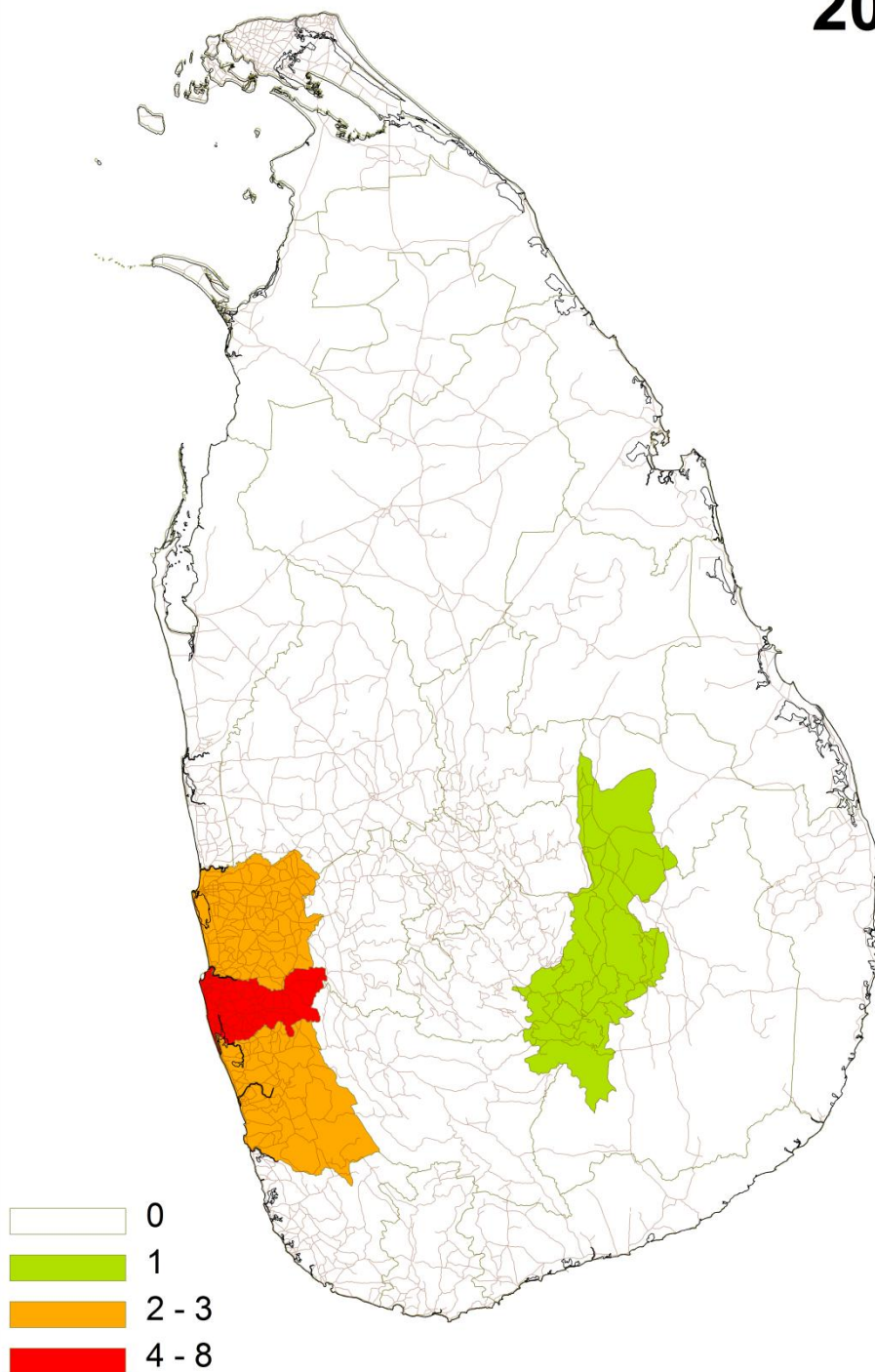

**2002**

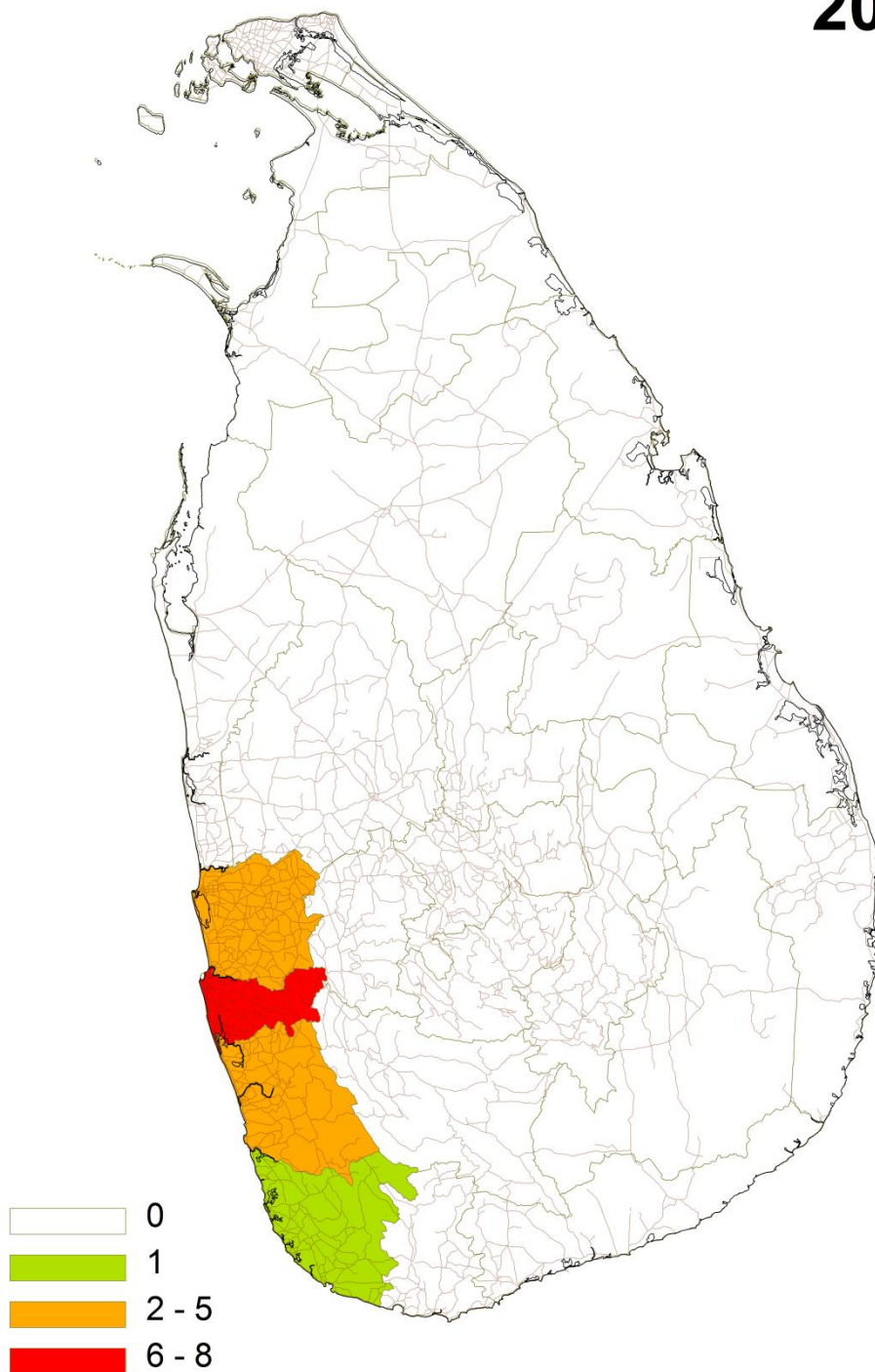

**2003**

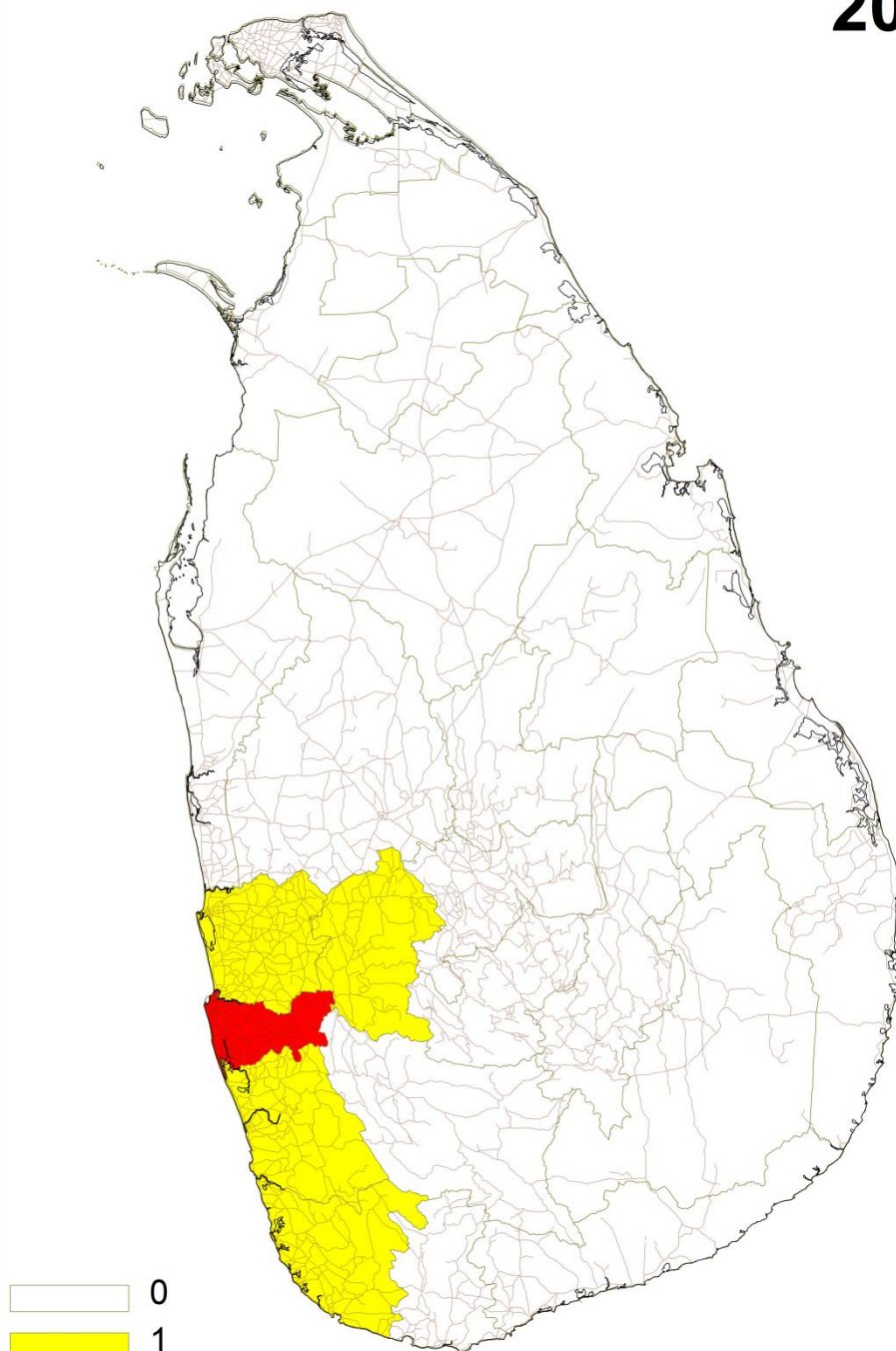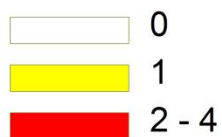

**2004**

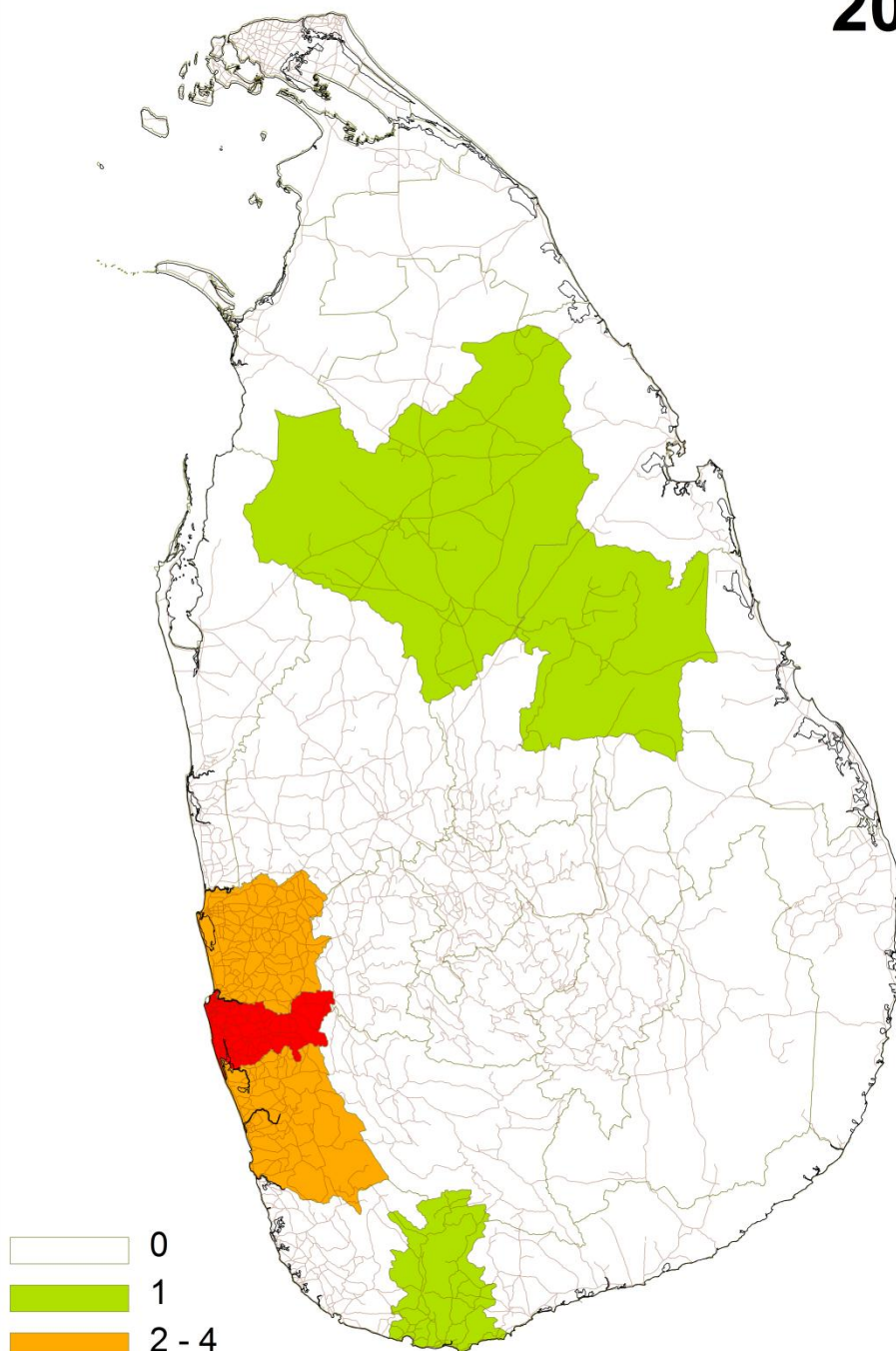

**2005**

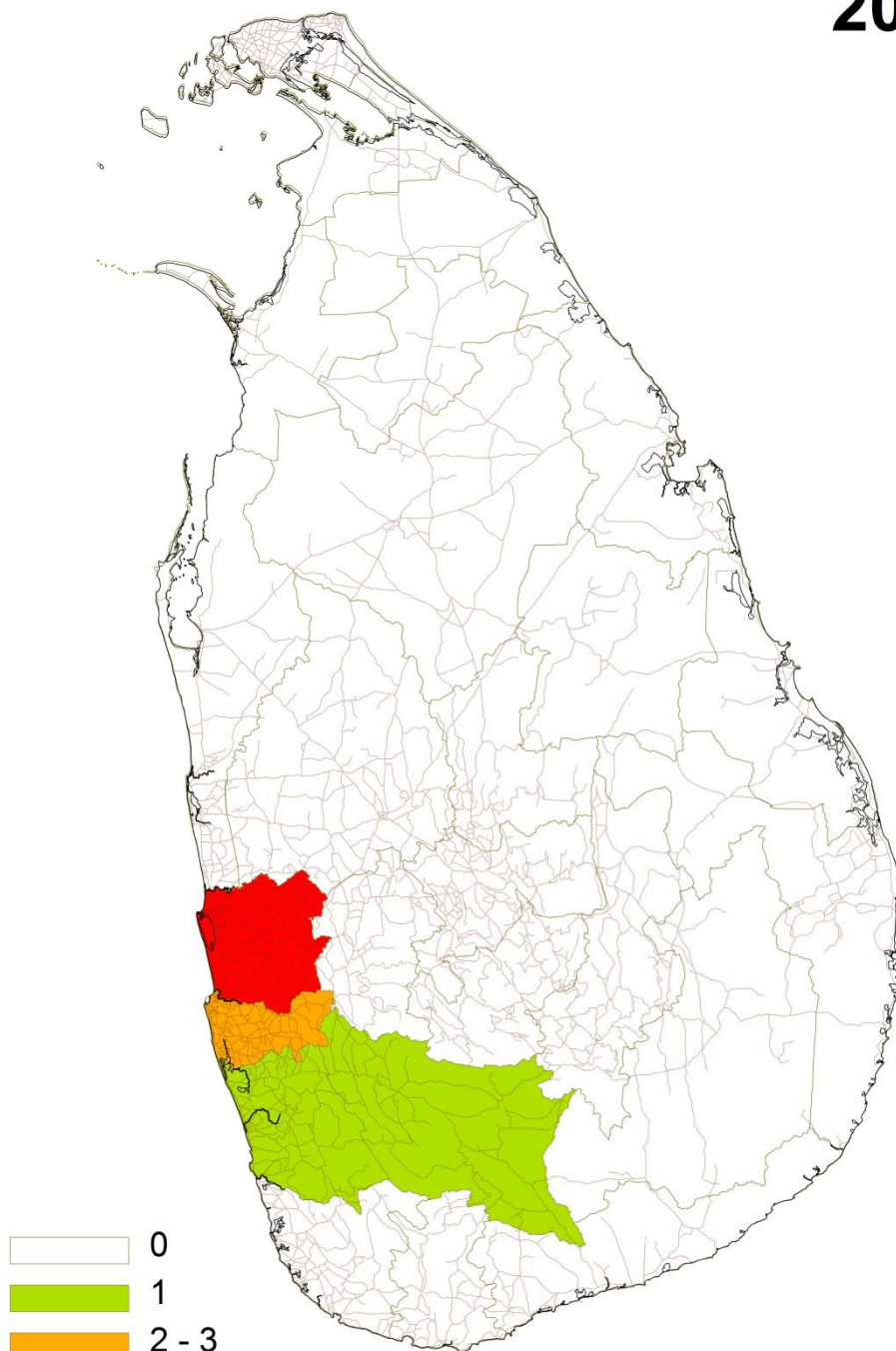

**2006**

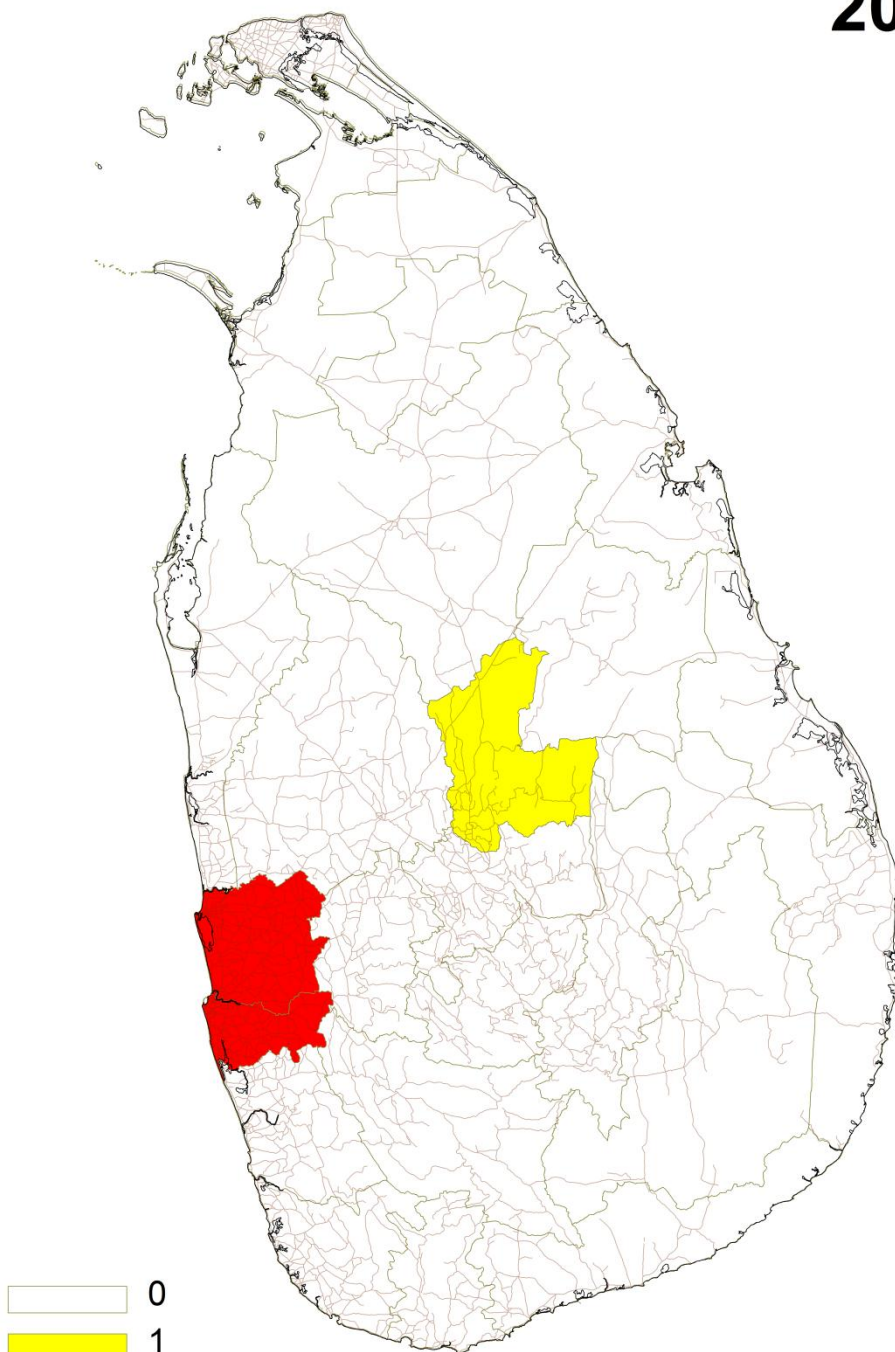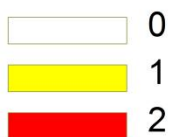

**2007**

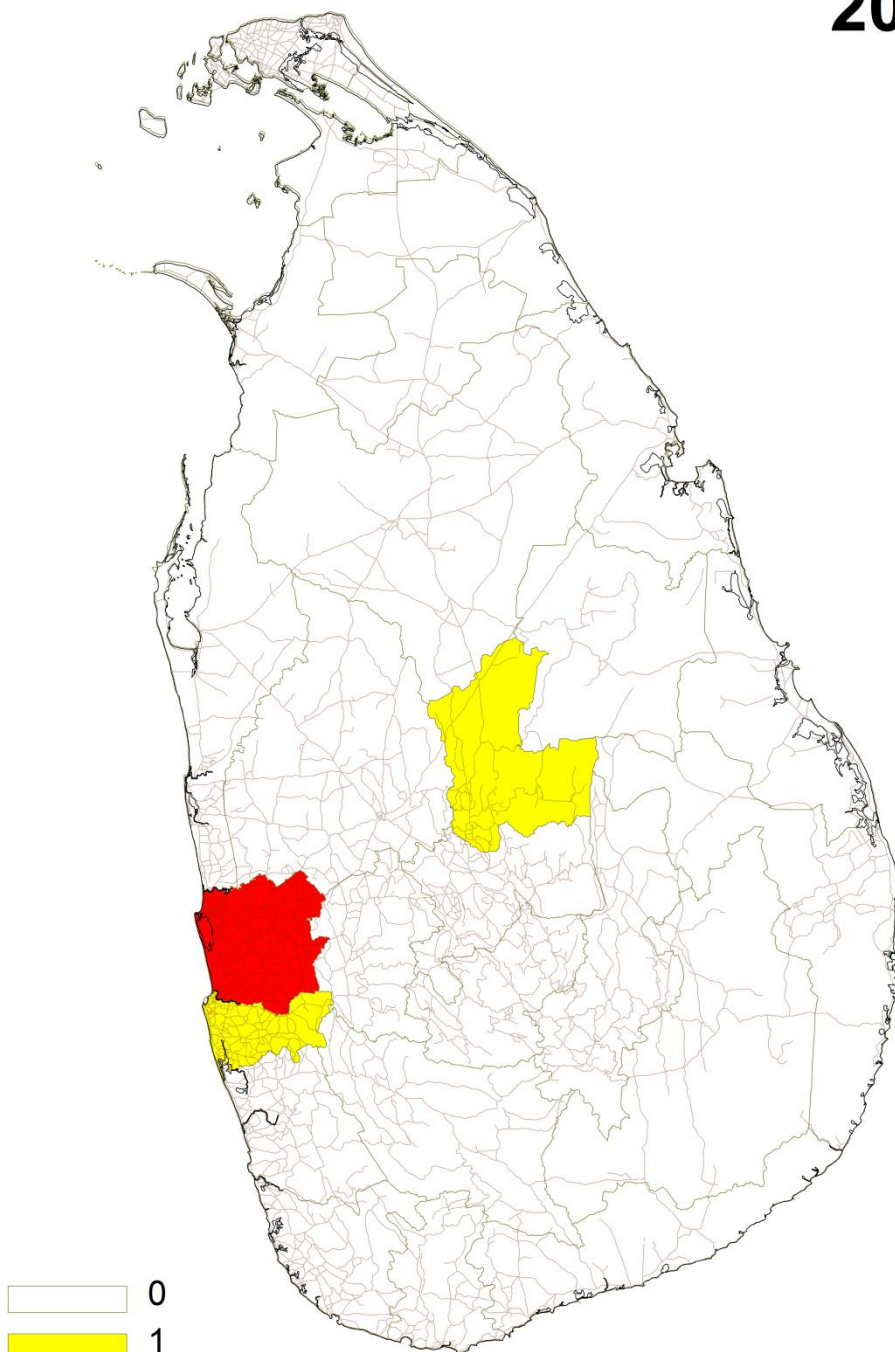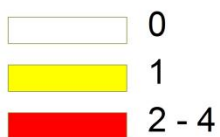

**2008**

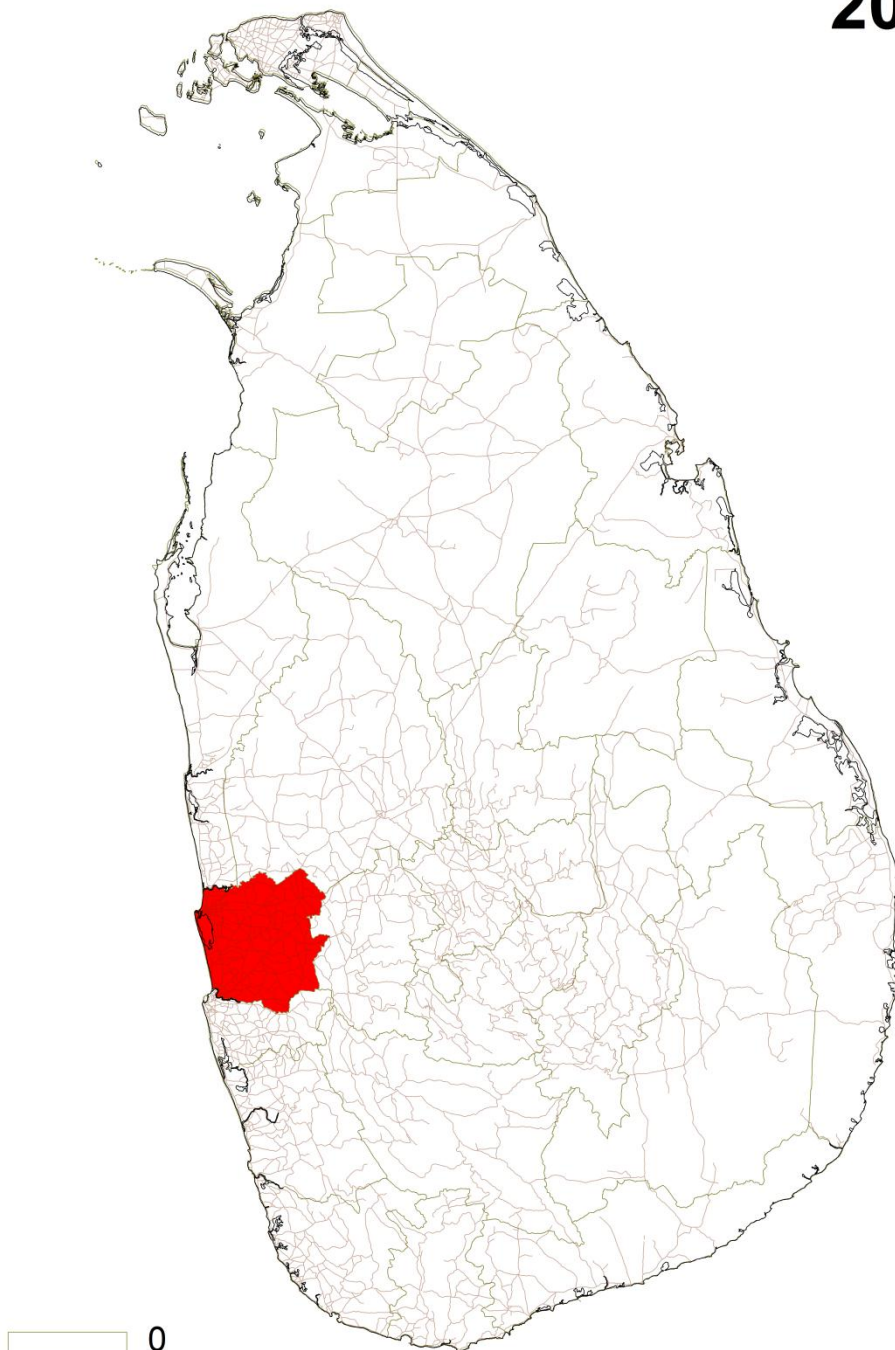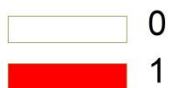

**2009**

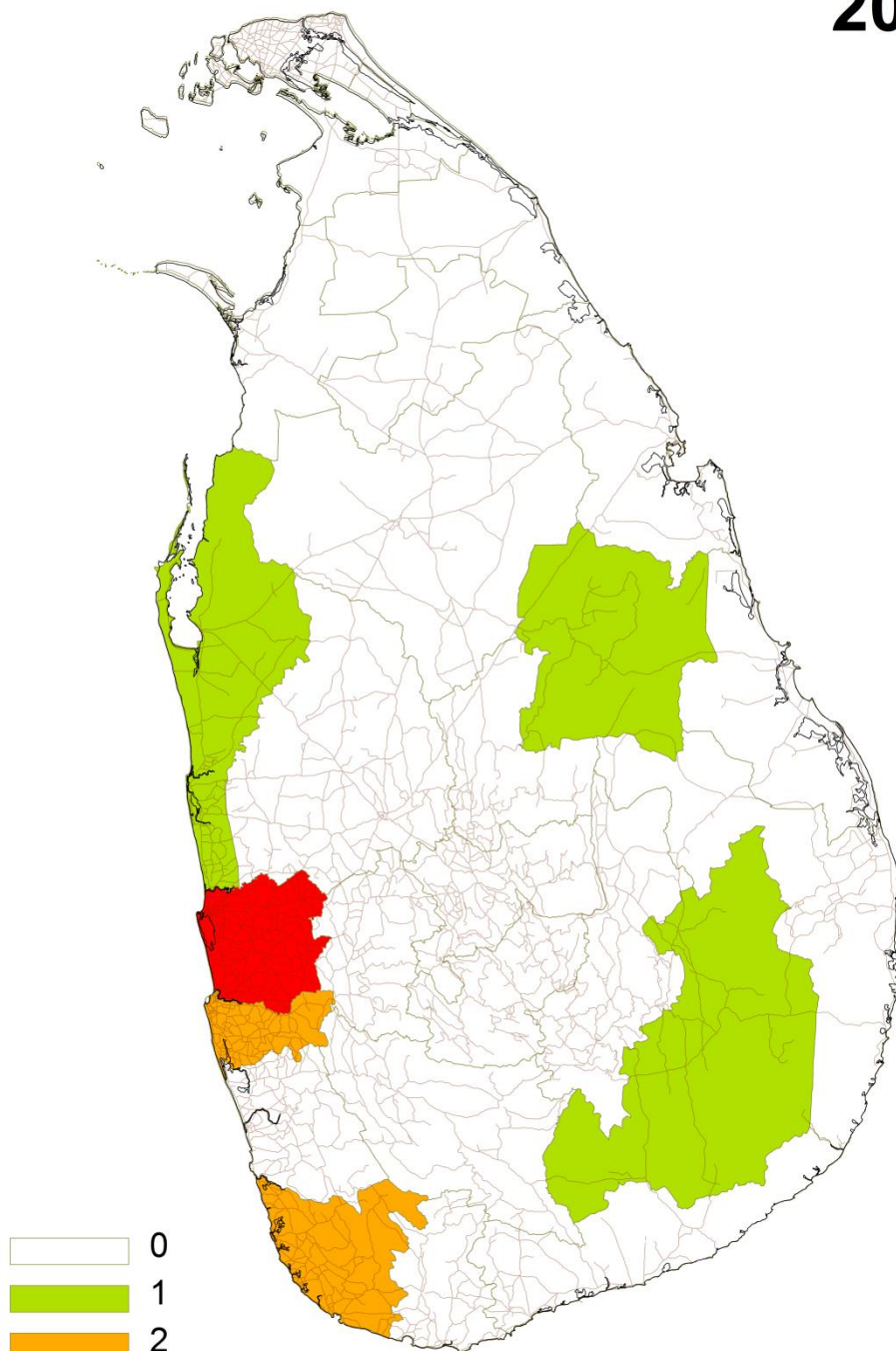

**2010**

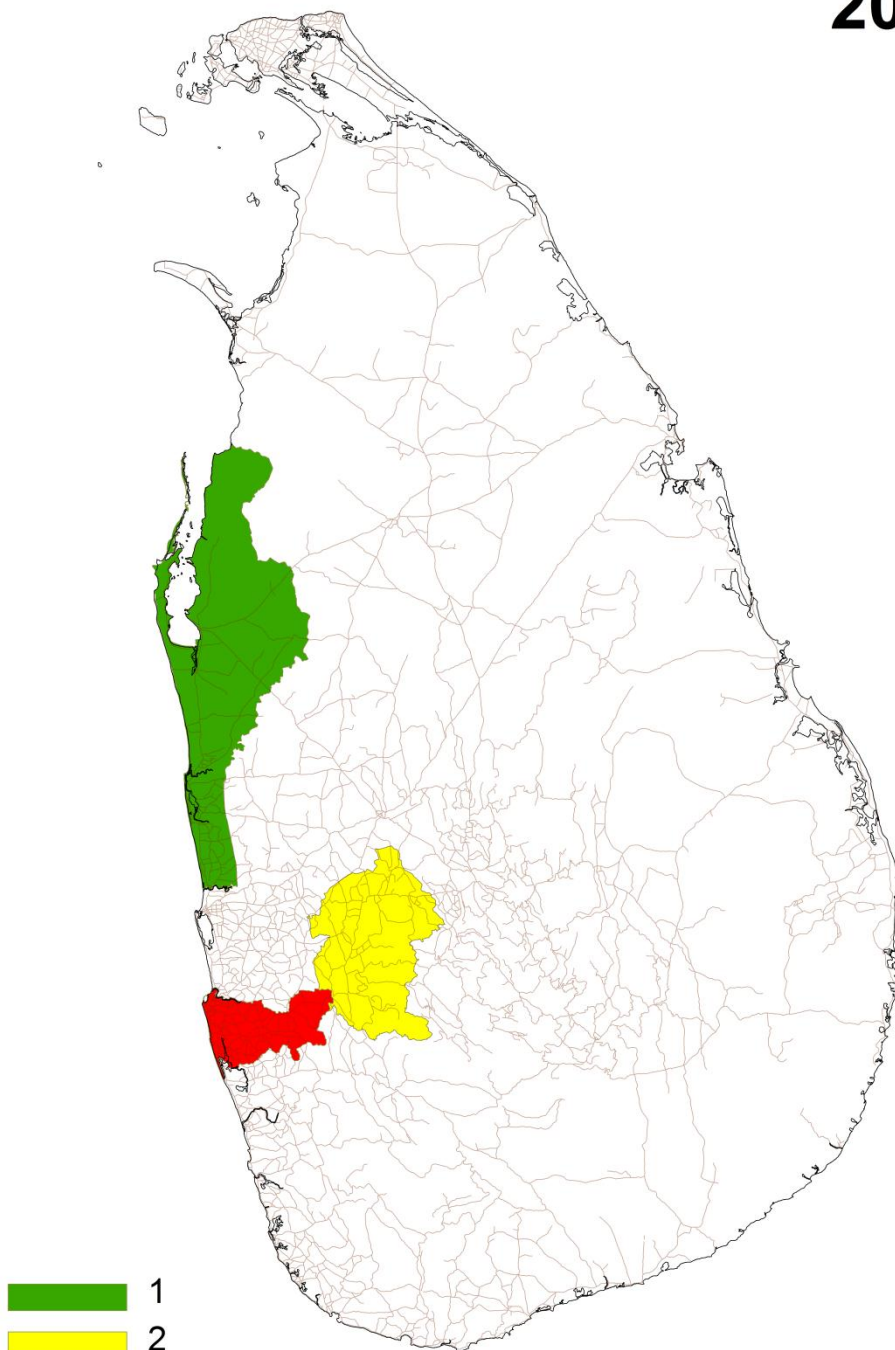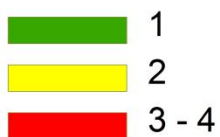

Supplement: Figure S2 — The distribution of wild animal rabies cases in different districts from 1999 to 2010. (PDF) [file pntd.0003205.s002.pdf]
